# Influence of Annealing Temperature on the OER Activity of NiO(111) Nanosheets Prepared via Microwave and Solvothermal Synthesis Approaches

**DOI:** 10.1021/acsami.4c14277

**Published:** 2024-11-01

**Authors:** Dereje H. Taffa, Elliot Brim, Konstantin K. Rücker, Darius Hayes, Julian Lorenz, Omeshwari Bisen, Marcel Risch, Corinna Harms, Ryan M. Richards, Michael Wark

**Affiliations:** † Institute of Chemistry, Chemical Technology I, Carl von Ossietzky University of Oldenburg, Carl-von-Ossietzky-Str. 9-11, 26129 Oldenburg, Germany; ‡ Department of Chemistry, Colorado School of Mines, 1500 Illinois St., Golden, Colorado 80401, United States; § Institute of Engineering Thermodynamics, German Aerospace Center (DLR), Carl-von-Ossietzky-Str. 15, 26129 Oldenburg, Germany; ∥ Nachwuchsgruppe Gestaltung des Sauerstoffentwicklungsmechanismus, Helmholtz-Zentrum Berlin für Materialien und Energie GmbH, Hahn-Meitner-Platz 1, 14109 Berlin, Germany; ⊥ Chemical and Material Sciences Center, National Renewable Energy Laboratory, Golden, Colorado 80401, United States

**Keywords:** electrolysis, oxygen evolution reaction (OER), microwave synthesis, supercritical solvent, electrocatalysts, surface termination, facet control, rock salt

## Abstract

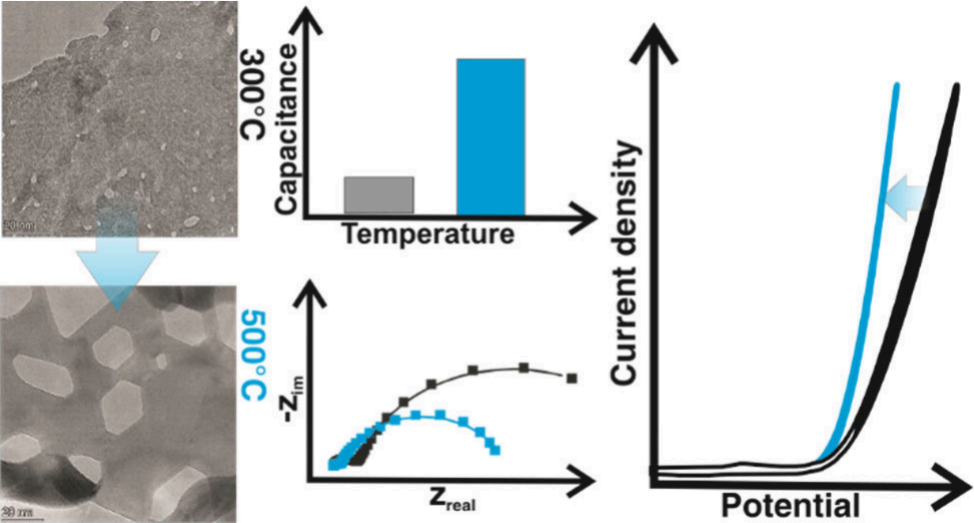

Earth-abundant transition metal oxides are promising alternatives to precious metal oxides as electrocatalysts for the oxygen evolution reaction (OER) and are intensively investigated for alkaline water electrolysis. OER electrocatalysis, like most other catalytic reactions, is surface-initiated, and the catalyst performance is fundamentally determined by the surface properties. Most transition metal oxide catalysts show OER activities that depend on the predominantly exposed crystal facets/surface structure. Therefore, the design of synthetic strategies to obtain the most active crystal facets is of significant research interest. In this work, rock salt NiO OER catalysts with (111) predominantly exposed facets were synthesized by a solvothermal (ST) method either heated under supercritical or microwave-assisted (MW) conditions. Particular emphasis was placed on the influence of the post annealing temperature on the structural configuration and OER activity to compare their catalytic performances. The as-prepared electrocatalysts are pure α-Ni hydroxides which were converted to rock salt NiO (111) nanosheets with hexagonal pores after heat treatment at different temperatures. The OER activity of the electrodes has been evaluated in 0.1 M KOH using geometric and intrinsic current densities via normalization by the disk area and BET area, respectively. The lowest overpotential at a geometric current density of 10 mA/cm^2^ is found for samples pretreated by heating between 400 and 500 °C with a catalyst loading of 115 μg/cm^2^. Despite the very similar nature of the catalysts obtained from the two methods, the ST electrodes show a higher geometric and intrinsic current density for 500 °C pretreatment. The MW electrodes, however, achieve an optimal geometric current density for 400 °C pretreatment, while their intrinsic current density requires pretreatment over 600 °C. Interestingly, pretreated electrodes show consistently higher OER activity as compared to the poorly crystalline/less ordered hydroxide as-prepared electrocatalysts. Thus, our study highlights the importance of the synthesis method and pretreatment at an optimal temperature.

## 1 Introduction

Material design for enhanced performance in electrocatalysis is approached either by increasing the number of active sites or by increasing the intrinsic activity of active sites.^1^ Control of the surface properties like the predominantly exposed crystal facets/surfaces can thus be used to increase the number of more active crystal facets if synthetic strategies are available for the respective catalyst materials. Transition metal oxides offer versatile material chemistry with varied redox states of the metal centers and adopted crystal structures. Among them, nickel-based oxides are intensively studied as oxygen evolution reaction (OER) electrocatalysts. Previous studies on thin films of NiO show the OER activities of different crystal facets following the order (110) > (111) > (100), which is attributed to the structural difference of the catalytically active hydroxides formed during the OER.^2^ A recent study of lanthanum nickelate perovskite thin films with different surface facets showed that the bulk facet orientation influences the electrochemical activity. Furthermore, the (111)-terminated surfaces favor the transformation to nickel oxyhydroxide, leading to lower overpotentials.^3^ However, most NiO reports on the facet-dependent activity studies use high-quality thin films developed on crystallographically oriented substrates using high vacuum techniques.^4,5^ For practical applications and to produce these materials in bulk, designing a solution based synthesis strategy is crucial. For rock salt type metal oxides, like NiO, the most thermodynamically stable surface is the (100) surface having the lowest formation energy.^6^ The Richards group successfully synthesized α-Ni(OH)_2_ nanosheets via a solvothermal method utilizing benzyl alcohol as a structure-directing agent followed by a subsequent supercritical drying step. Upon controlled thermal treatment steps, the hydroxides were converted into NiO (111) nanosheets with hexagonal pores, which act as a cation intercalating host structure.^7,8^ In contrast, Lu et al. reported a hydrothermal approach to synthesize β-Ni(OH)_2_, which led to NiO nanosheets with dominant (111) orientations.^9^ Straightforward comparison of the facet-dependent activity requires control of further morphological properties such as the particle size that could also influence the catalytic performance. Therefore, developing alternative and versatile synthesis strategies for the metastable (111) and (110) surfaces would facilitate a fundamental study of the relationship of the preparation and ultimate properties of materials.

Microwave synthesis (MW) plays a very important role in synthetic organic chemistry and has been shown to be one of the most sought-after synthetic approaches in the development of new functional materials.^10,11^ MW synthesis relies on the efficient heating of reaction media (precursors and solvent) by microwave dielectric heating, which, in turn, involves the absorption of microwave energy by the media and the ability to convert it into heat. MW heating is achieved mainly through two main processes: dipolar polarization and ionic conduction.^12,13^ Both heating mechanisms work when the dipole and polar units of the reactants (ions) in the reaction mixture interact with the microwave electric field at a specified frequency. The dipoles/ions constantly seek to align with the oscillating electric field, leading to molecular friction, which ultimately generates heat (dielectric loss). The amount of heat produced is directly related to the ability of the media to interact with the microwave field frequency. This direct and local heating effect leads to several synthetic advantages compared to conventional heating methods: this includes significantly reduced reaction times and fast reaction rates, selective heating resulting in high selectivity of products, and enhanced control of the reaction parameters.

Owing to the above synthetic advantages, MW synthesis demonstrates its power for the development of diverse electrocatalyst systems, for which the OER electrocatalysts take the vast majority. Accordingly, various MW-prepared OER electrocatalysts were reported including supported metal nanoparticles,^14−16^ metal oxides/hydroxides,^17−20^ and metal organic frameworks (MOFs).^21,22^ Among the metal oxides/hydroxides, Ni oxides/hydroxides gained a significant interest as a potential replacement of the highly OER active precious metal-based catalysts. Several studies have shown that the OER activities of pristine Ni oxides/hydroxides are highly dependent on the size, shape, and morphologies of the prepared materials.^23,24^ Furthermore, crystal orientation (exposed facets) greatly influences the OER activities of transition metal oxides as the different facets display dissimilar polarity, surface energies, and adsorption properties.^25,26^ Hence, identifying the most active crystal facets for the OER and designing a synthetic approach for the same is intensively researched in the pursuit of finding OER-active yet earth-abundant materials.^3,26,27^

In this study, the synthesis of α-Ni(OH)_2_ nanosheets using fast MW synthesis is reported in comparison to solvothermally prepared materials. A subsequent controlled annealing step converts the hydroxides to rock salt NiO (111) nanosheets. Furthermore, the OER activities of both materials after annealing at different temperatures were explored at low current density. The results suggest that the two synthetic approaches led to structurally similar catalyst materials with modest morphological variations. However, the optimal OER activities are observed at different annealing temperatures due to the variations in morphology and the catalytically relevant electrochemical active surface area (ECSA). The present work demonstrates a facile way of preparing NiO (111) nanosheets via MW routes with the potential to be extended to other facets and incorporation of additional transition metal ions to boost the OER activity, which will be studied in future work.

## 2 Experimental Section

### 2.1 Chemicals

Analytical grade chemicals (>99.0%) were used as received without further purification: Nickel(II) nitrate hexahydrate [Ni(NO_3_)_2_·6H_2_O] (Sigma-Aldrich), urea [NH_2_CONH_2_](Sigma-Aldrich), benzyl alcohol [C_6_H_5_CH_2_OH] (Sigma-Aldrich), and methanol pure [CH_3_OH] (Fisher Chemical). NiO nano powder (US-nano, US Research Nanomaterials Inc., USA), micron-sized NiO powder (Carl Roth, Germany), and LiNiO_2_ (Sigma-Aldrich, Germany) were used as commercial standards.

### 2.2 Microwave Preparation of NiO (111) Nanosheets

**Scheme 1:**
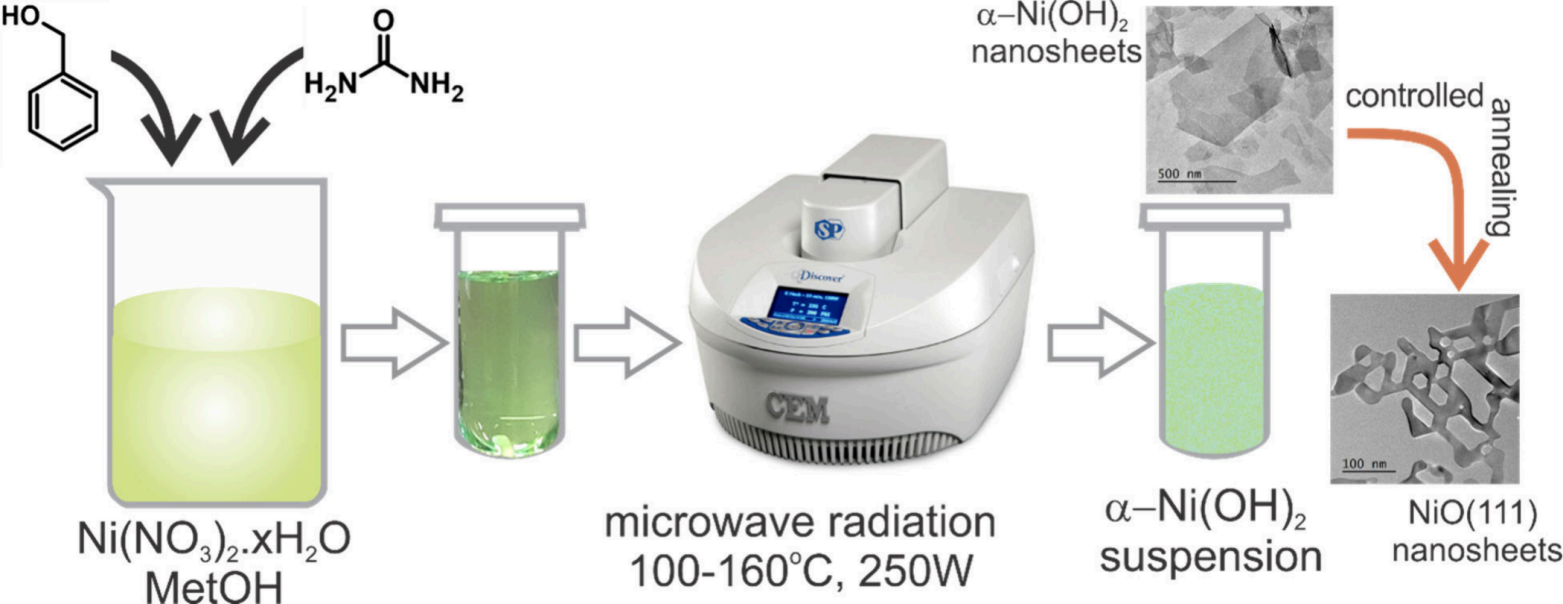
Major Synthesis Steps Involved in the MW Preparation of NiO (111) Nanosheets with Hexagonal Pores

NiO (111) nanosheets were prepared following a modified procedure reported for the solvothermal synthesis.^7^ Briefly, 0.30 mol of Ni(NO_3_)_2_·6H_2_O (1.75 g) was dissolved in 20 mL of pure methanol and stirred to obtain a light green solution. Then 0.15 mol of urea (0.18 g) was added to the solution and further stirred for 10 min. Subsequently, 0.6 mol of the benzyl alcohol (1.23 g) was added, and the resultant solution was transferred into a 35 mL microwave glass vial. The reaction was carried out at 140 °C for 30 min under stirring using the microwave synthesizer Discover SP (CEM Corporation, USA). During the synthesis, the pressure reached 10–12 bar. Afterward, the reaction mixture was cooled with an air circulation system to room temperature. The obtained nanosheets were repeatedly washed with pure methanol to remove the unreacted reagents and dried at 60 °C overnight in a vacuum oven at 250 mbar. The nanosheets were subsequently calcined in a box furnace (Linn High Therm, Germany) at different temperatures ranging between 300 and 600 °C for 3 h at a heating and cooling rate of approximately 3 °C min^–1^. The resulting calcined nanosheets changed their color from black at low temperature to dark gray at temperatures between 400 and 500 °C, and above 600 °C, they turned to light gray. The microwave synthesis parameters, such as the synthesis time, temperature, and heating rate were optimized. Additionally, different ratios of Ni:urea were tested, and their influences on the morphologies were investigated (Scheme 1). Solvothermal synthesis of NiO (111) nanosheets was carried out following a reported protocol.^7^

### 2.3 NiO (111) Nanosheets Prepared by the Solvothermal Method

The NiO (111) nanosheets were synthesized by the solvothermal route followed by pseudo-supercritical solvent removal as described elsewhere.^7^ In brief, Ni(NO_3_)_2_·6 H_2_O, urea, and benzyl alcohol were combined (ratio 2:1:4), dissolved in 50 mL of methanol with stirring for 1 h, and then transferred to a 600 mL autoclave. The system was purged with Ar for 1 min and then pressurized to 9 bar. The autoclave was heated to 265 °C (pseudo-supercritical point of methanol solution) and held for 90 min, and finally the methanol vapor was released, yielding a green powder.

### 2.4 Characterization

#### 2.4.1 Physicochemical Characterization

The phase purity and crystallinity of the prepared nanosheets were studied by using powder X-ray diffraction (PXRD). The PXRD of the samples was measured using an Empyrean Series 2 diffractometer (PANanlytical, Netherlands) with Cu Kα radiation (λ = 0.154 nm). The PXRD patterns were recorded in Θ–2Θ configuration between 5° and 80° 2Θ. Raman measurements were performed on a Senterra Raman microscope (Bruker) equipped with a 488 nm laser source. The power was set to 10 mW with a spot size of 1 μm, except the α-Ni(OH)_2_, which was measured at 5 mW. The integration time was set to 20 s, and two coadditions were measured between 0 and 2600 cm^–1^.

The surface composition and chemical state of the materials were analyzed by X-ray photoelectron spectroscopy (XPS) with an ESCALAB 250 Xi (Thermo Fischer, England) with a monochromic Al Kα X-ray source (1486.6 eV). The measurements were recorded at a pass energy of 10 eV with a step size of 20 meV. The adventitious carbon C 1s peak at 284.8 eV is used as a charging reference. The spectra were analyzed using the Avantage software version 4.97 with a Shirley background.

Transmission electron microscopy (TEM) measurements were performed with an FEI Talos F200x TEM operating at 200 kV for structural determination through lattice plane spacing. NiO nanosheets were dispersed in ethanol under ultrasonication and drop-cast on copper grids with carbon spports. High-resolution TEM images were taken from an 8 μm spot size with 200 ms exposure time.

Nitrogen adsorption–desorption isotherms were recorded at −196 °C with a Tristar II adsorption setup (Micromeritics, USA). Prior to the measurement, the annealed nanosheets were degassed at 150 °C for 4 h, while as-prepared nanosheets were degassed at 90 °C overnight. Isotherms are collected between 0.005 and 0.95 *p*/*p*_0_ relative pressure, and the BET surface areas were analyzed following the Brunauer–Emmett–Teller (BET) approach, while the pore size distribution was estimated using the BJH (Barrett–Joyner–Halenda) method.

X-ray absorption (XAS) measurements at the Ni K-edge were collected at the KMC-2 beamline at the BESSY II electron storage ring (300 mA, top-up mode) operated by Helmholtz-Zentrum Berlin für Materialien and Energie.^28^ Further details of the experimental setup are described elsewhere in detail.^29^ To prepare the samples, a thin and uniform layer of the powder was spread on a Kapton tape. After the surplus powder was removed, the tape was folded multiple times to create 1 cm × 1 cm windows. The MW-NiO (111)-500 and ST-NiO (111)-500 samples, along with commercial reference Ni-oxides (please refer to Figure S5 for XRD and BET data), were measured in transmission mode using ion chambers. The XAS energy was calibrated by setting the first inflection point of a simultaneously measured Ni foil to 8333 eV. Normalization of all spectra involved the subtraction of a straight line obtained by fitting the data before the K-edge and division by a polynomial function obtained by fitting the data after the K-edge. The Fourier transform (FT) of the extended X-ray absorption fine structure (EXAFS) was determined between 35 and 550 eV (3 to 12 Å^–1^) above the K-edge, with *E*_0_ values set at 8333 eV for Ni.

Atomic force microscopy (AFM) experiments were performed at an NX-10 (Park Systems) microscope with a HiRes-C14/Cr-Au cantilever (MikroMasch) having a nominal resonance frequency of 160 kHz, force constant of 5 N m^–1^, and tip radius of <1 nm. The shown images were acquired with a scan frequency of 0.4 Hz at 512 × 512 pixels in true non-contact mode (Park Systems). The samples were prepared from catalyst suspensions like the TEM experiments but were coated onto p-type (boron) silicon wafer substrates that were previously cleaned with ultrapure water.

#### 2.4.2 Electrochemical OER Activity Measurements

NiO (111) powder inks were prepared using 1.8 mL of water, 0.2 mL of 2-propanol, 9.0 μL of 5% Nafion solution (Nafion D520CS 1000 equiv, Ion Power), and 4 mg of the different NiO (111) powders. The inks were sonicated using a titanium ultrasonic horn Q500 sonicator (QSONICA, USA) for 5 min. As working electrodes, 3 mm diameter glassy carbon (GC) electrodes (Metrohm, Germany) were used. The electrodes were polished sequentially with alumina powder slurries of 1 and 0.05 μm particle diameter each for 1 min, respectively. To clean the polished electrodes, they were sonicated in pure water and isopropanol for 5 min each.

Then 4 μL of the inks was dropped onto a prepolished glassy carbon electrode and spin coated at a rotation speed of 750 rpm for 30 min to reach a catalyst loading of ∼115 μg cm^–2^. After air-drying, the electrodes were ready for use.

The NiO (111)-coated electrodes were mounted in the rotating disc electrode (RDE) assembly (AUT.RDE.ROT.S, Metrohm, Germany), and OER activity and electrochemical double layer capacitance (*C*_DL_) measurements were conducted in a custom-made single-compartment Teflon cell. The graphite rod counter and Hg/HgO reference (ALS Co. Ltd., Japan) electrodes were separated from the main solution with homemade salt bridges. A 0.1 M KOH solution is used as electrolyte. Prior to each set of electrochemical tests, the electrolyte solution was bubbled first with N_2_ for at least 30 min and kept under a N_2_ atmosphere. *C*_DL_ measurements were first run in a potential range of 0.90–1.15 V vs RHE where minimal faradaic current response was observed to estimate the ECSA. Cyclic voltammetry (CV) measurements were conducted in stationary solution by sweeping the potential from more negative to positive potential and back at different scan rates: 5, 10, 25, 50, 100, 250, and 500 mV s^–1^. The working electrode potential was held at each potential vertex for 20 s before the next scan.

For the OER activity measurements, the solution was bubbled with O_2_ for 10 min and maintained under an O_2_ atmosphere. To access the initial OER activities, three scans of a rotating disk electrode voltammetry were recorded at 2500 rpm by sweeping the potential from 1.14 to 1.94 V vs RHE at a scan rate of 10 mV s^–1^. Then, the electrodes were activated by 50 CV cycling steps in the potential range 1.04–1.64 V vs RHE at a scan rate of 100 mV s^–1^. Following this activation step, three CV cycles were recorded to evaluate the final OER activities. To determine the uncompensated solution resistance (*R*_u_) and charge transfer resistance for the OER reaction, electrochemical impedance spectroscopy (EIS) measurements were performed in the frequency range of 1 Hz to 100 kHz with 10 mV AC amplitude. All electrochemical measurements are carried out with an Autolab PGSTAT12 potentiostat (Metrohm, The Netherlands) controlled by NOVA software (version 2.1). The post thermal treatment and activity measurement protocols were kept the same for a fair comparison of samples based on the two synthesis approaches.

## 3 Result and Discussion

Generally, the formation of α-Ni(OH)_2_ is favored at pH values lower than 8, and the presence of urea is crucial in maintaining the pH. The reaction involves, first, the thermal decomposition of urea to release NH_3_, which later undergoes slow hydrolysis to form NH_4_^+^ and OH^–^ ions. These ions gradually react with Ni^2+^ to form α-Ni(OH)_2_ and control the crystal growth.^30,31^ The XRD patterns of the selected MW-synthesized hydroxides are presented in Figure S1a,f, all prepared at 140 °C, showing the effect of MW synthesis time. All samples show high crystallinity, where as short as 15 min MW synthesis time is enough to induce crystalline hydroxides; their crystallinity and yield increase as the synthesis time increases. The measured diffraction peaks correspond very well to a pure hexagonal structure of α-Ni(OH)_2_ with a *P*3̅1*m* space group (PDF 00-038-0715) and can be indexed to those reported for crystalline α-Ni(OH)_2_.^32^ The sample exhibits major peaks at 2θ values of 11.98°, 23.33°, 33.32°, and 59.31°, which correspond to the (001), (002), (110), and (300) crystal planes, respectively. Notably the two diffraction peaks at low 2θ degrees shift as the MW synthesis time increases, and the largest shift observed for samples synthesized at 180 min and the ST sample (Figure S1). Previous reports showed that the diffraction peak representing the (001) plane is related to the interlayer distances between the nanosheets and is affected by the size and number of ions and solvent molecules incorporated within the layers.^33,34^ As the synthesis is carried out in methanol with nitrate-containing metal precursors, both methanol and nitrate ions are expected to be present. The interlayer distance between the sheets slightly decreases (2θ shift to larger value) with the MW synthesis time, and the shift is significant for supercritically dried ST samples resulting from stacked nanosheets (Supporting Information Figure S1). A similar trend is observed as the MW temperature increases from 120 to 160 °C. Following these results, a MW synthesis time of 30 min and a temperature of 140 °C was chosen as standard synthesis conditions for further investigations.

**Figure 1:**
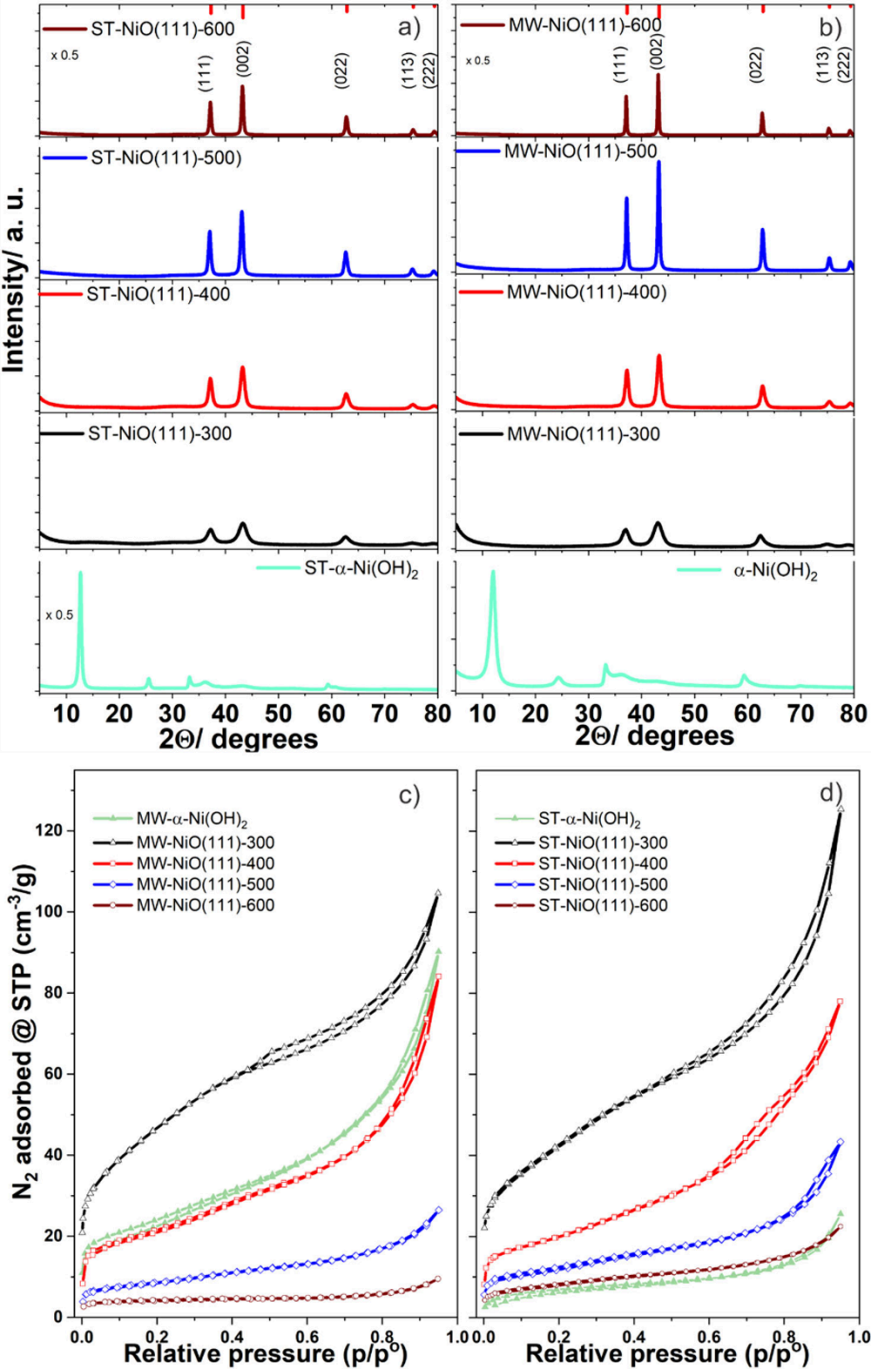
(a) Powder XRD patterns of NiO(111) nanosheets heat treated at different temperatures: (a) MW prepared and (b) ST prepared. Line patterns correspond to the reference (PDF 98-018-4918). Corresponding N_2_ gas adsorption isotherms: (c) MW and (d) ST prepared NiO(111) nanosheets.

The influence of controlled thermal treatment on the structural and phase composition of the samples was investigated by annealing the samples between 300 and 600 °C for 3 h. The powder XRD patterns with the respective annealing temperature are presented in Figure 1a and b. The diffraction patterns show that synthesized hydroxides with an α-Ni(OH)_2_ structure are converted to the pure rock salt NiO without impurity phases. The XRD patterns exhibit primary diffraction peaks centered at 2θ values of 37.13°, 43.2°, 62.72°, 75.29°, and 79.23° assigned respectively to (111), (002), (220), (311), and (222) planes of face-centered-cubic (fcc) type NiO with a space group of *Fm*3*m* (PDF 98-018-4918). The higher the annealing temperature, the narrower and sharper the respective diffraction peaks are, which suggests increasing crystallite size and higher crystallinity. For both synthesis methods, slight shifts of the reflexes to lower 2θ values were observed with increasing annealing temperature, indicating lattice contraction. It is worth mentioning that the relative ratios of the integral intensity of the (111) planes to the (002) planes slightly decreased for both samples with increasing temperature (Figure S2a). To demonstrate the (111) oriented nanosheets growth, the MW samples were directly grown on glass substrates by inserting a cleaned glass substrate in the MW reactor. Indeed, after annealing, the samples show higher integral intensity ratios of (111) planes to (002) planes, suggesting the NiO (111) is dominantly present (Figure S2b).

The average crystallite sizes for both the MW and ST samples were calculated using the Scherrer equation.^35,36^ The crystallite sizes range between 4 and 49 nm for the MW samples and between 6 and 25 nm for the ST samples, respectively (Table 1). Compared to the MW samples, the ST samples exhibit smaller crystallite sizes within the temperature windows of this study.

**Table 1:** Summary of the Results Obtained from XRD and BET Analysis^a^

	BET area (m^2^/g)		Crystallite size (nm)		BJH pore size (nm)	
Annealing *T* (°C)	MW	ST	MW	ST	MW	ST
-	67.2 ± 0.2	25.3 ± 0.3	-	-	3.0	-
300	165 ± 1.4	149.6 ± 1.0	4.4	6.0	2.9	3.0
400	75.9 ± 0.1	70.5 ± 0.2	11.0	10.2	3.5 (6.8)	3.4 (8.5)
500	30.2 ± 0.1	41.6 ± 0.1	26.3	17.9	3.4	3.4
600	15.4 ± 0.2	28.2 ± 0.1	49.2	25.3	-	3.0

a MW: microwave and ST: solvothermal.

To obtain further structural insights, Raman spectra of the samples were collected (Figure S3a and b). Generally the α-Ni(OH)_2_ shows low intensities due to the crystalline disorder/amorphous nature of the samples. The oxide samples show Raman scattering signals characteristic for NiO.^37,38^ All oxide samples show dominant bands between 200 and 500 cm^–1^ that are assigned to the one-phonon transverse-optical (TO) mode. Another signal between 700 and 1200 cm^–1^ is assigned to two-phonon processes, and the intensity increases with higher calcination temperatures. A well-developed one-phonon peak is mostly associated with surface effects and defects which are usually absent in single-crystalline samples.^39^ For the MW-NiO(111) samples, the two phonon peak centered at 1000 cm^–1^ is more pronounced at higher temperatures, but for ST-NiO(111) the signal intensity remains unaffected. The presence of this band indicates the crystalline nature of the temperature-treated samples and agree with the XRD results.^39^

Functional properties, including catalysis and charge storage, benefit from high surface area materials. The surface area and porosity characteristics of the nanosheets were investigated through nitrogen adsorption–desorption measurements shown in Figure 1c and d. Isotherms of samples annealed at relatively low temperature show significant N_2_ uptake at low relative pressures, suggesting that the NiO(111) nanosheets possess micropores. The isotherms exhibit typical type-II isotherms with hysteresis loop type H4 characteristics indicating materials with platelet-like structures.^40^ The relative pressure at which the hysteresis loop started to close shifted to a higher relative pressure as the annealing temperature increased. This suggests the formation of larger pores. BET and BJH methods were used to calculate the specific surface area and pore size distribution of NiO(111) nanosheets, respectively. The as-prepared MW α-Ni(OH)_2_ has a specific area of 66 m^2^/g compared to the 25 m^2^/g for the ST α-Ni(OH)_2_. The highest specific surface area is achieved for samples annealed at 300 °C with 165 and 149 m^2^/g for the MW and ST samples, respectively. The specific surface area decreased as the temperature increased further to 15 and 28 m^2^/g at 600 °C for the MW and ST samples, respectively. Note that for the MW samples, the specific surface area drops more with temperature than for the ST samples. The loss of surface area with temperature is related to the coarsening of the crystals or coalescence of the micropores to form larger macropores (Table 1). The BJH pore size distribution shows a first maximum centered at around 3 nm for most samples annealed below 500 °C for MW samples, suggesting that the nanosheets mainly possess micropores. Interestingly, the ST and MW samples both annealed at 400 °C also show a second maximum indicating larger pores of ∼8 and ∼7 nm diameter, respectively (Figure S4). For the ST samples the micropore structure is preserved up to 600 °C.

**Figure 2:**
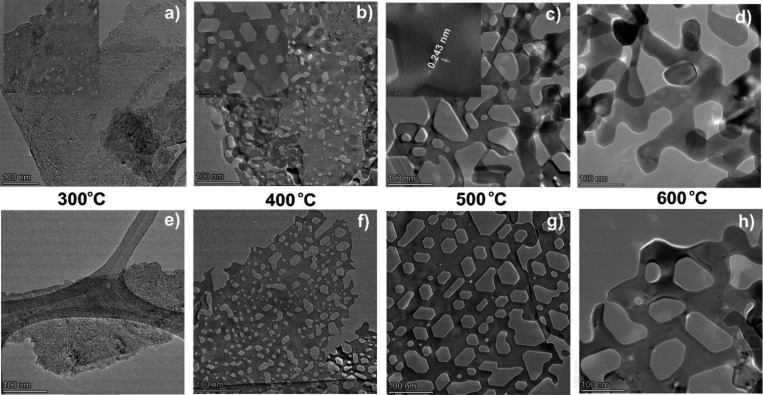
HR-TEM images (a–d) for MW samples and (e–h) for ST samples showing the formation of hexagonal pores on annealing of the samples. The measured lattice spacing suggests the NiO(111)-oriented nanosheets.

To investigate the structural and morphological similarities or differences of the NiO(111) samples, we conducted TEM measurements (Figure 2). The synthesized α-Ni(OH)_2_ exhibits nanosheet morphology with layer dimensions in the sub-micrometer range (Figure S6). On low-temperature treatment (300 °C), small pores of random shapes appear on the nanosheets. The width of the pores varied from 1.7 to 3.1 nm. The sizes are in good agreement with the values obtained in the BJH pore size distribution. As the annealing temperature increases to 400 °C, well-defined hexagonal pores are formed with their width ranging from 10.0 to 15.0 nm and the length from 20.0 to 30.0 nm. Note that there are also smaller hexagons with 5.0 to 6.0 nm widths, leading to the observed second maximum in the BJH analysis. After heating to 500 °C, hexagonal pores with widths of 15.0 to 20.0 nm and lengths of 25.0 to 30.0 nm were observed, these hole sizes being too large to be observable by N_2_ adsorption. Larger pores of irregular sizes are observed, probably due to the merging of smaller hexagons. The nanosheet structure is lost at 600 °C, and plate-like crystals are formed. For the ST samples, defined hexagonal pores of various sizes started to form at 400 °C and remained stable for higher temperatures. HR-TEM images show highly crystalline NiO nanosheets predominantly oriented in the (111) plane. The measured lattice fringes at 500 °C give wavelengths of 0.243 and 0.240 nm for the MW and ST samples, respectively. These values correspond well to the reported (111) lattice plane. The size and density of pores were estimated by using scanning transmission electron microscopy (STEM) in high-angle annular dark-field imaging (HAADF) mode (Figure S7). Generally, the ST samples display larger pores compared to those of the MW samples. Interestingly, however, the total fractional area covered by the pores is higher for the MW samples than for the ST samples. Such detailed morphological variations may lead to different OER activities. Sun et al. showed that the edges and corners of the hexagonal pores have a (001) orientation, which are catalytically more active than the flat basal (111) plane of the nanosheets.^41^

To further gain morphological insights, noncontact mode AFM studies were performed to estimate the thickness of the nanosheets (Figure S8). Samples annealed at 400 °C show a comparable thickness of 12 and 14 nm for the ST and MW samples, respectively. The results further strengthen the evidence that the two materials have similar dimensions in 3D space. Visible pores in the ST sample annealed at 400 °C compared to the MW sample where no pores are visible underline the observations in HAADF-STEM that the ST sample possesses larger pores compared to the MW sample.

To investigate the surface composition and oxidation states of Ni species, XPS investigations were conducted. The survey spectra of both samples show only C, O, and Ni as main components (Figure S9). The synthesized hydroxide samples and the samples treated at 300 °C contain N species originating from either unreacted urea or metal nitrate precursors used in the synthesis (Figure S10). As the MW samples underwent rigorous washing steps, the amount of N is very low compared to the ST samples, which are supercritically dried.

**Figure 3:**
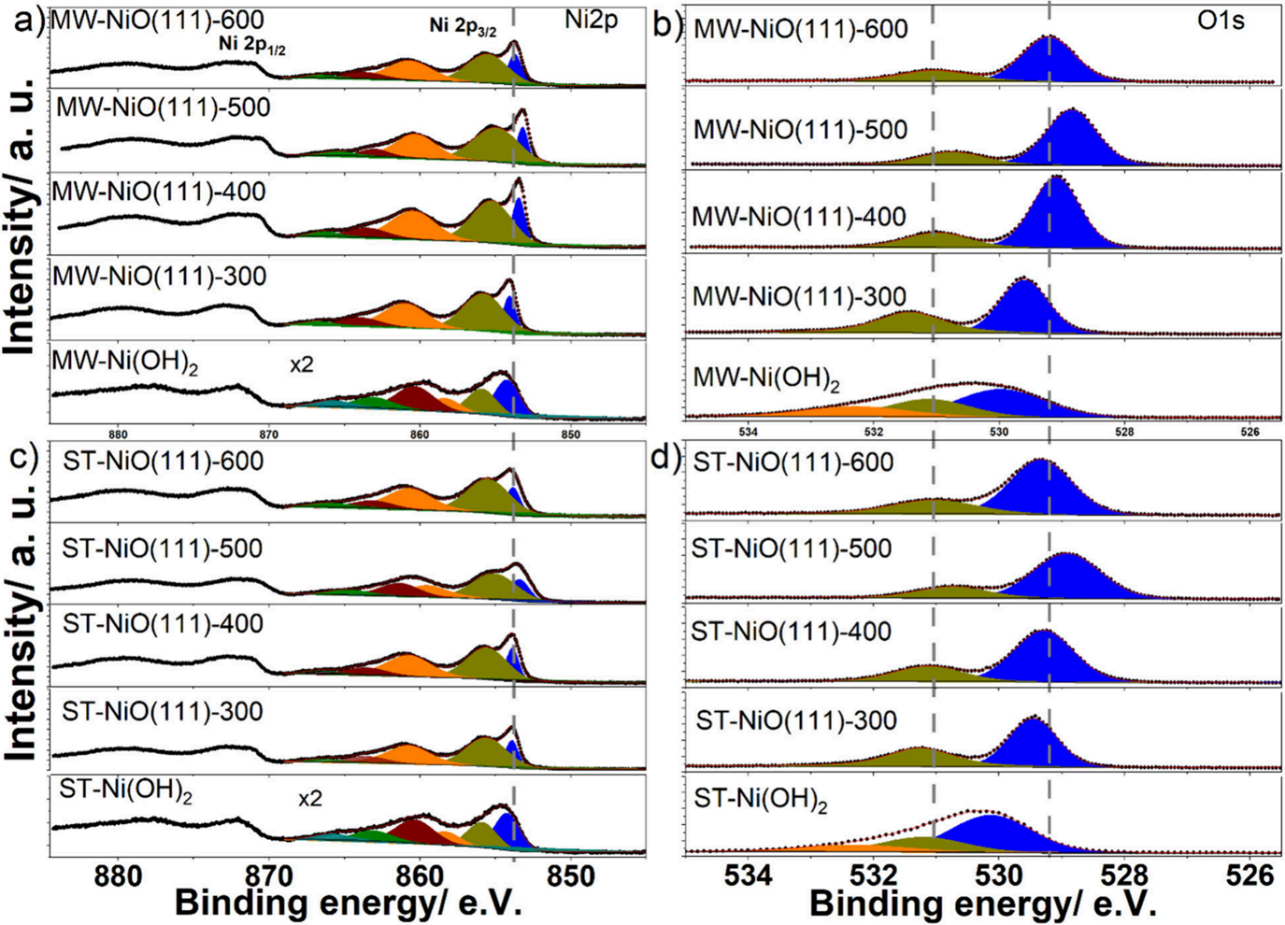
XPS Ni 2p (a, c) and O 1s (b, d) spectra for MW and ST NiO(111) nanosheets annealed at different temperatures. For the as-prepared samples, three O 1s components are observed. For the temperature-treated samples, only two peaks are observed. The broadness of the Ni 2p emission decreases with increasing temperature.

The O 1s spectra (Figure 3b and d) of all annealed samples mainly presented two emission lines. The binding energies of the main lines vary between 528.9–529.6 eV and 528.9–529.5 eV for the MW and ST samples, respectively, and correspond to lattice oxygen (Ni–O–Ni) in NiO.^42^ This line shifts generally to lower binding energies as the temperature increases, which may be due to the formation of crystalline NiO with lower numbers of surface hydroxide groups. The position of the second emission line varies between ∼531.0 and 531.3 eV and between 531.0 and 531.4 eV for the ST and MW samples, respectively. The second line is attributed to either to Ni-OH or oxygen defects in the lattice structure.^43^ For the prepared samples before thermal treatment, the O 1s spectra feature one additional line at higher binding energies of 532.3 and 532.2 eV for the ST and MW samples, respectively. These peaks are assigned to absorbed water or C–O species. Carbonate species are likely to be present as the samples contain a relatively high amount of C (Figure S10).

The Ni 2p spectra show complex spectral features and are split into Ni 2p_3/2_ and Ni 2p_1/2_ doublet domains due to the spin–orbit coupling.^44^ Ni 2p core level spectra of the MW and ST samples, which were annealed at different temperatures, are shown in Figures 3a and c. The Ni 2p_3/2_ line shifts from 854.2 eV for the as-prepared hydroxides to 853.3 eV after annealing at 600 °C for the MW samples and from 854.2 to 853.4 eV for the ST samples in the same annealing temperature window. The binding energy values are consistent with earlier reports and show higher binding energies for Ni-hydroxides than NiO species.^42,45^ Beisinger et al. reported binding energy values of 854.9 and 853.7 eV for the Ni 2p_3/2_ line for the Ni(OH)_2_ and NiO, respectively.^42,44^ Hence, the measured values in the current study suggest that Ni species are in the +2 oxidation state near the surface.

As the temperature is raised from 300 to 600 °C, the surface Ni:O ratios of the annealed samples decrease from 0.97 to 0.85 and 0.95 to 0.81 for the MW and ST samples, respectively (see Table S1). The ratios are smaller than the expected value of 1, suggesting oxygen-rich surfaces. Previous theoretical and experimental studies showed that the (111) surface is the most polar one among the NiO surfaces and is OH terminated.^46,47^ Hence, the observed lower ratios of Ni/O at the surface agree with these reported studies. Such high coverage of O atoms consequently may lead to nonstoichiometry at the surface or the formation of oxidized surface atoms, in this case Ni^3+^. However, the quantification of the Ni^3+^ has proven to be challenging due to the very strong satellite feature of Ni 2p, which complicates the separation of nonlocal screening and surface effects from the vacancy-induced Ni^3+^ ion.^48,49^

**Figure 4:**
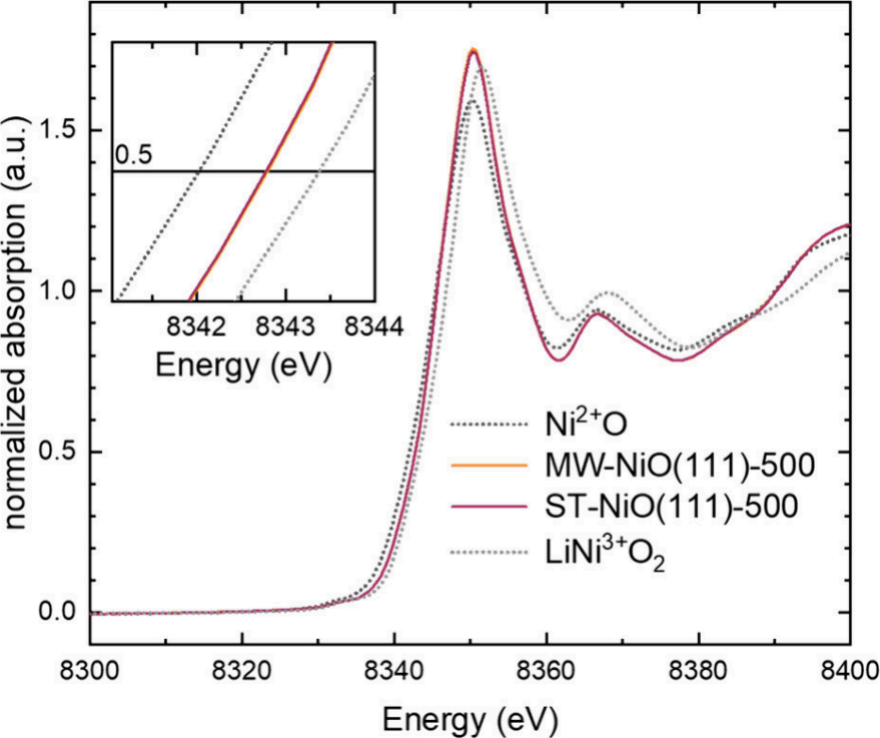
XANES spectra at the Ni K-edge showing the edge energy of the MW-NiO(111)-500 and ST-NiO(111)-500 samples with commercially available standards NiO and LiNiO_2_.

X-ray absorption spectroscopy (XAS) provides information related to the bulk oxidation state of the materials and the coordination environment of the atoms in the lattice structure.^50^ In Figure 4, the Ni K-edge X-ray absorption near-edge structures (XANES) of MW-NiO(111)-500 and ST-NiO(111)-500 are presented and compared with the two commercial reference samples NiO (see Figure S5 for XRD and BET data) and LiNiO_2_ with a nominal Ni oxidation state of +2 and +3, respectively. The Ni K-edge at 0.5 normalized intensity of the MW-NiO(111)-500 and ST-NiO(111)-500 samples overlapped and was between those of the references (inset of Figure 4), indicating the two samples have similar bulk oxidation states near +2.5. Bulk oxidation was also found for the commercial nanosized NiO (Figure S11b), and the same charge redistribution (reduced surface, oxidized bulk) was also found, e.g., in LiMn_2_O_4_ nanoparticles.^29,51^ The Fourier transform of the EXAFS of MW-NiO(111)-500 and ST-NiO(111)-500 exhibits the two prominent peaks corresponding to the Ni–O and Ni–Ni coordination path, as shown in Figure S11a. Similar peak position and shape associated with MW-NiO(111)-500 and ST-NiO(111)-500 signifies no observable structural differences, which is further supported by XRD.

All of the above results suggest that the two sets of samples (MW and ST) have closely resembled electronic structures but with few distinct morphological and crystal size differences.

**Figure 5:**
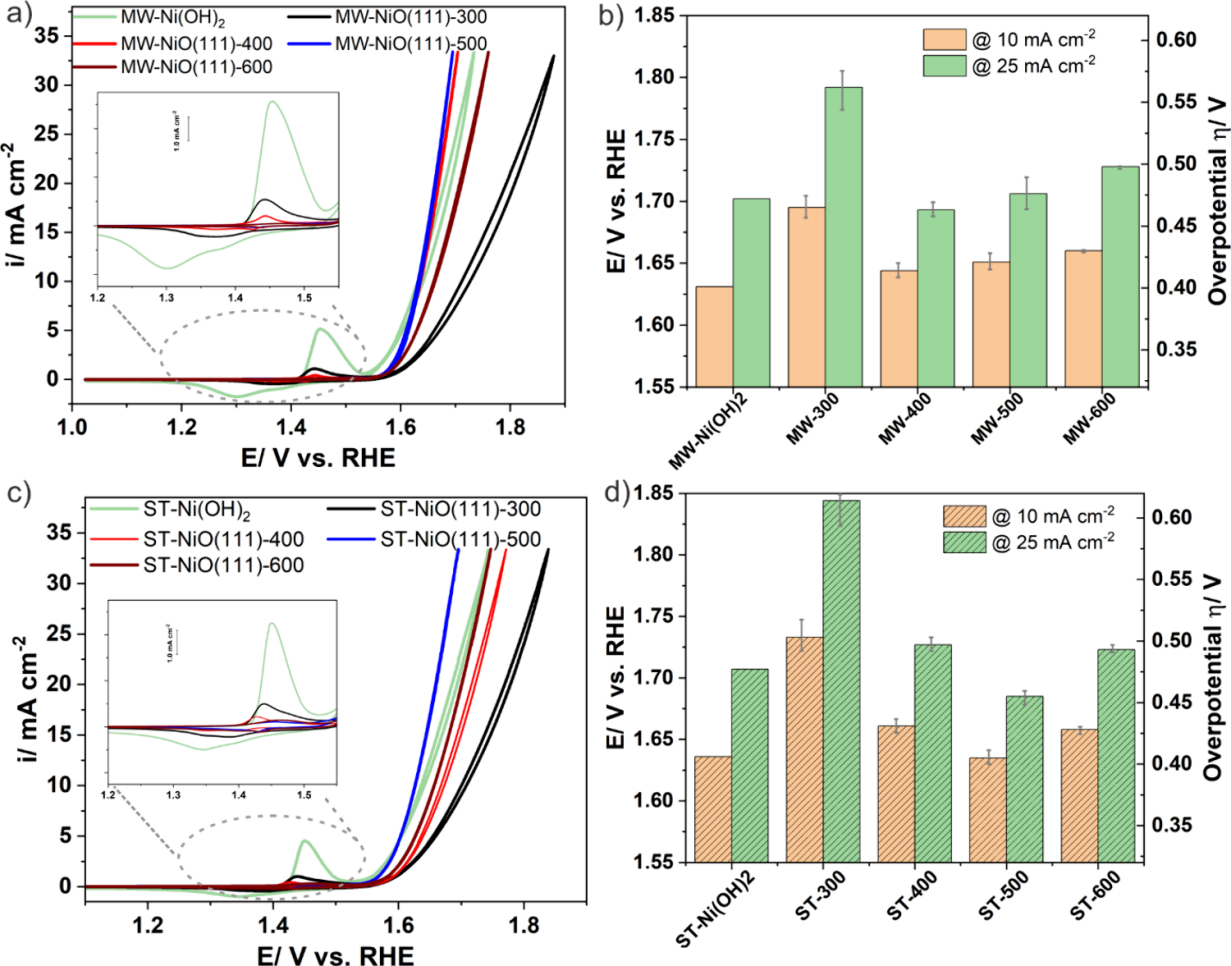
(a) *iR*-corrected cyclic voltammograms of (a) MW-NiO(111) nanosheets and (c) ST-NiO(111) nanosheets, annealed at different temperatures and measured at 10 mV s^–1^ in 0.1 M KOH solution. The insets show the Ni^2+/3+^ redox transitions. Corresponding overpotential comparison at 10 mA/cm^2^ and 25 mA/cm^2^ of (b) MW-NiO(111) nanosheets and (d) ST-NiO(111) nanosheets.

To determine their catalytic behavior, we conducted electrocatalytic OER activity tests in a standard three-electrode configuration under hydrodynamic conditions using an RDE setup in alkaline media. Our measurement protocol includes measurements of the double capacitance (*C*_DL_) in a potential window (0.90–1.15 V vs RHE) where no Faradaic reactions take place, followed by a conditioning step to activate the electrodes and a final activity test in the OER region. The *C*_DL_ of both MW and ST samples before and after activation are shown in Figures S12 and S13, respectively. Generally, the *C*_DL_ decreases after activation, and among the activated samples the MW-NiO(111)-500 and ST-NiO(111)-500 samples show the highest *C*_DL_. However, these values should be treated with care, as reliable capacitive curves are difficult to obtain for low conductive metal oxides like NiO annealed at low temperatures and some curves show ohmic behaviors.^52^ Prior to the OER activity measurements, the electrodes are conditioned (activated) in 0.1 M KOH by recording 50 consecutive cyclic voltammograms in the potential window between 1.15 and 1.65 V vs RHE (Figure S14). The voltammograms reveal two characteristic features: a redox wave centered at 1.40 V vs RHE followed by a steep increase in current visible at higher potentials. These are well-known behaviors of Ni oxy/hydroxides in alkaline electrolytes attributed to the transformation between Ni^2+^(OH)_2_ and Ni^3+^OOH, with the subsequent oxidation current due to the oxygen evolution in alkaline electrolytes.^37,53^ The current for the NiO(111) nanosheet modified electrodes increases gradually and reaches a relatively stable value after 40 scans. The gradual increase of Ni^2+/3+^ redox peak current suggests the conversion of the dense NiO rock salt surface to the more electrolyte permeable oxy/hydroxides and conductive structures as previously reported.^54^ Unlike NiO, the respective Ni(OH)_2_ shows relatively stable CVs during the activation period (Figure S15). The MW-NiO(111)-modified electrodes show a narrow redox peak, but the corresponding peak for ST-NiO(111) modified electrodes is broad. This difference may originate from variations in catalyst coating (poor catalyst–electrode contact) or an inherent electrical conductivity difference of the two nanosheets. For both samples, the first scan is anodically shifted compared to the subsequent scan. Additionally, the comparison of the CVs before and after the activation step for the samples clearly shows that there is improved activity, which is further reflected in terms of redox peak current and the onset potential of the OER (Figure S16). Such behavior was also previously reported for NiO electrocatalysts; this phenomenon is related to the formation of the more active Ni oxy/hydroxides (NiOOH) or the segregation of Fe from the NiOOH phase if the electrolyte solutions contain Fe impurities.^45,55−57^ In order to check the Fe impurities in the electrolyte, we have conducted ICP–OES analysis (Thermo Scientific iCAP PRO series) of the electrolyte, which prove the absence of Fe impurities or, if present, a concentration below the detection limit of 0.105 μg/L. To evaluate the OER activities of the electrodes, we have performed three consecutive CVs after the activation step. The third scan of each series is shown in Figures 5a and c. For all activated samples, the Ni^2+/3+^ redox transition is observed (insets). A closer look at the samples reveals that hydroxide samples show a more positively shifted redox wave. Previous reports confirmed that both NiO(111) and α-Ni(OH)_2_ form γ-NiOOH on their surfaces during the OER.^2^ Thus, the electrochemically formed surface hydroxides on the NiO(111) nanosheets and α-Ni(OH)_2_ are similar, and the difference in the CV behavior most probably relies on the different electronic structure of the bulk materials. The MW samples show a more symmetric and intense wave, and the anodic charge for Ni^2+/3+^ transition follows the order MW-Ni(OH)_2_ > 300 °C > 400 °C > 500 °C > 600 °C. Similarly for the ST samples the order is ST-Ni(OH)_2_ > 300 °C > 400 °C > 500 °C > 600 °C. The higher anodic peak charge can be interpreted as an indication of the presence of more electrochemically accessible Ni centers and is thus a parameter related to the surface area. Interestingly, our suggestion relates well to the decreasing trend of the integrated charge with increasing temperature, as presented in Figure S17, and it follows the BET area. The OER activity trend (discussed below) follows neither the BET area nor the charge order, indicating that not all electrochemically accessible Ni centers are active for the OER as previously. This observation agrees with reported trends on similar electrocatalytic systems.^58,59^

To compare the OER activity of the NiO(111) nanosheet electrodes, we calculated the overpotential values at 10 and 25 mA/cm^2^ current densities (normalized for the geometric area of the GC disc) for loading of ∼115 μg/cm^2^ (Figure 5b and d). As shown in Figure 5b, the lowest overpotential is observed at 400 and 500 °C for the MW and ST samples, respectively. To reach the 10 mA/cm^2^ current density, the MW-NiO (111) requires 414 mV overpotential, whereas the ST-NiO (111) needs 405 mV overpotential at the optimum annealing temperature. The overpotential values reached 463 and 455 mV to achieve a current density of 25 mA/cm^2^ for the most active MW and ST samples, respectively. The values are comparable or even slightly lower than the reported NiO nanoparticulate-based OER catalysts^53,60^ but are higher than values reported for NiO thin films at 10 mA/cm^2^ current density.^37^ Note that the OER activity trend of the samples does not match with the BET areas; that is, samples with high BET areas (treated at low temperature) display low OER activities. This could be attributed to the low crystallinity of the samples and the organic residues that block the catalytic centers from accessing the electrolyte. The trade-off between the crystallinity and the BET area is presumably achieved in the temperature window 400–500 °C, which shows high OER activity. These samples possess well-defined hexagonal cavities with varying sizes, as shown in Figure 2, characterized by additional edge sites. Previous studies revealed that the edges of these cavities demonstrate more distinct crystallographic orientations (100) than the basal plane and exhibit higher catalytic activity.^41^

**Figure 6:**
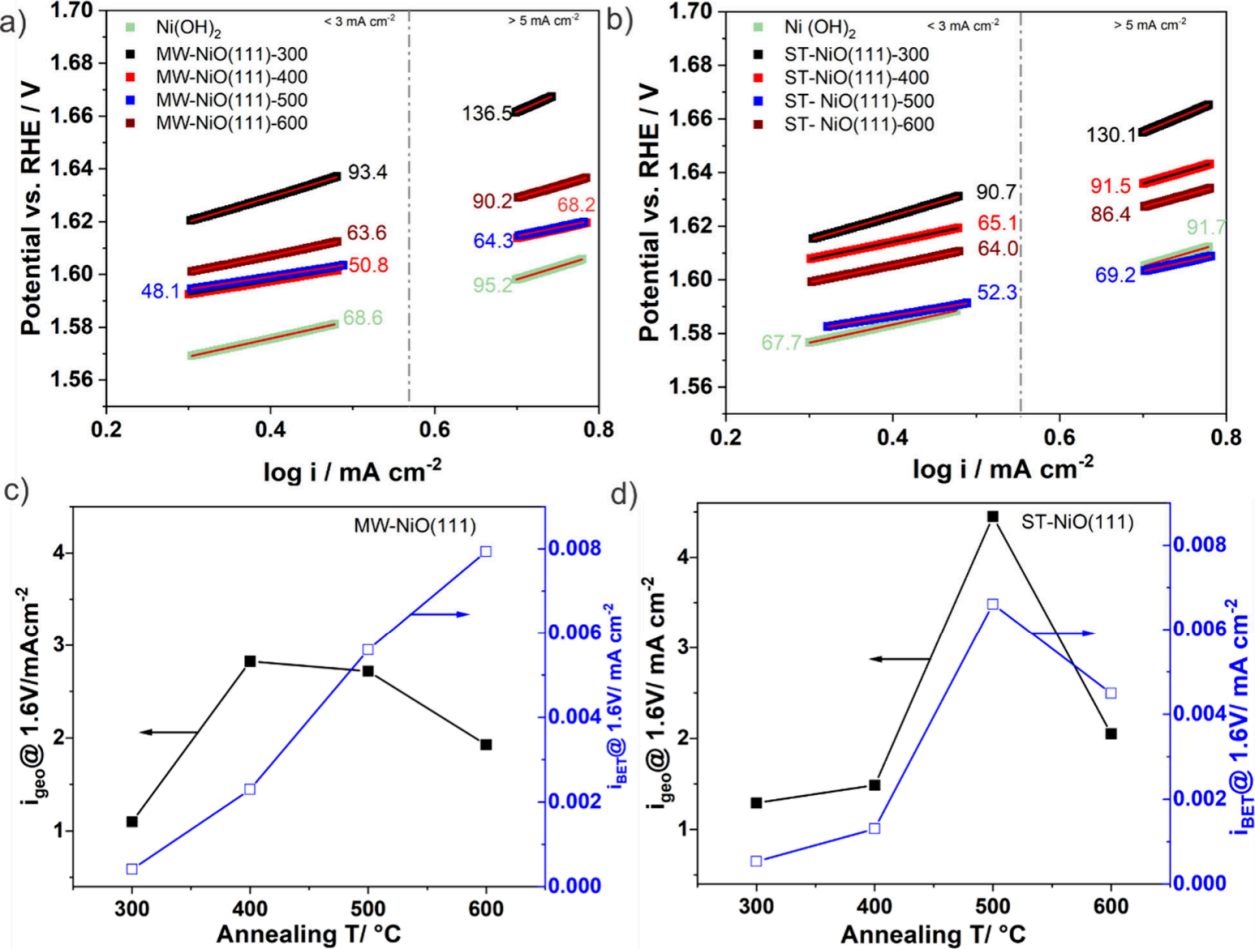
Tafel slopes analysis for low and high current density regions: (a) MW-NiO(111) and (b) ST-NiO(111) samples. Solid lines represent linear fits. Comparison of geometric area and BET area normalized current densities against temperature: (c) MW-NiO(111) and (d) ST-NiO(111) samples.

To gain more insight into the activity differences and the electron transfer kinetics at NiO(111)/electrolyte interfaces, Tafel slopes were extracted from the CVs shown in Figure 5a and c. The corresponding Tafel slopes are presented in Figure 6a and b. Generally, we identified two types of Tafel slopes depending on the current region chosen for the analysis. For the low current range <3 mA/cm^–2^ relatively small Tafel slopes are obtained. Tafel slopes of 50.8 and 52.3 mV dec^–1^ were calculated for the most active MW-NiO(111) and ST-NiO(111) samples, respectively. On the other hand, the Tafel slopes increase (vary between 63.6–136.5 mV and 67.7–130.1 mV for the MW and ST samples, respectively) when current ranges of >5 mA/cm^–2^ were used for the analysis, suggesting low OER activities. Our observation is consistent with other reported Tafel slope trends on NiO systems, and such difference could arise from different reaction pathways for the OER or the same reaction pathway but a change of the rate-determining step.^2,61,62^ Lower Tafel slopes are desirable, but further work is needed to optimize the samples for higher current densities. Interestingly, the Tafel slope reaches its minimum value between the temperature range of 400–500 °C.

To relate the observed seemingly similar activity trends of the two catalysts with their intrinsic material properties, the currents at a potential of 1.6 V were normalized with the BET area and compared with the geometric current density (Figure 6c and d). The trend showed that the geometric area normalized current densities (*i*_geo_) increased with the annealing temperature and reached their maxima at 400 and 500 °C for the MW-NiO(111) and ST-NiO(111) samples, respectively. But further temperature increases lead to a decrease in current density. Generally, the ST-NiO(111) samples show *i*_geo_ values higher than those of the MW-NiO(111) counterparts. On the other hand, the BET area normalized current densities (*i*_BET_) of the ST-NiO(111) samples show similar trend to the *i*_geo_ but keep increasing for MW-NiO(111) samples above 400 °C. Notably, BET is a common and accurate method to estimate the total surface area of a material, but the amount of the catalytically active sites is likely a fraction of the total area. Thus, the number of catalytically active sites may increase as the temperature increases despite the decrease in the total BET area. However, the coarsening or sintering of the nanosheets at elevated temperatures may lead to loss of active sites, and this phenomenon occurred at above 500 °C for the ST-NiO(111) samples. The MW-NiO(111) samples’ intrinsic activities (current densities normalized to BET area) could be improved until it reached the maximum value using higher annealing temperatures of >600 °C. Other suitable ECSA determination methods like oxygen adsorbate capacitance from EIS and AFM will be considered for future studies.^63^ An alternative explanation is that the intrinsic (average) activity of each active site increases with improved crystallinity as reported previously,^64^ which is compatible with increasing BET-normalized currents.

**Figure 7:**
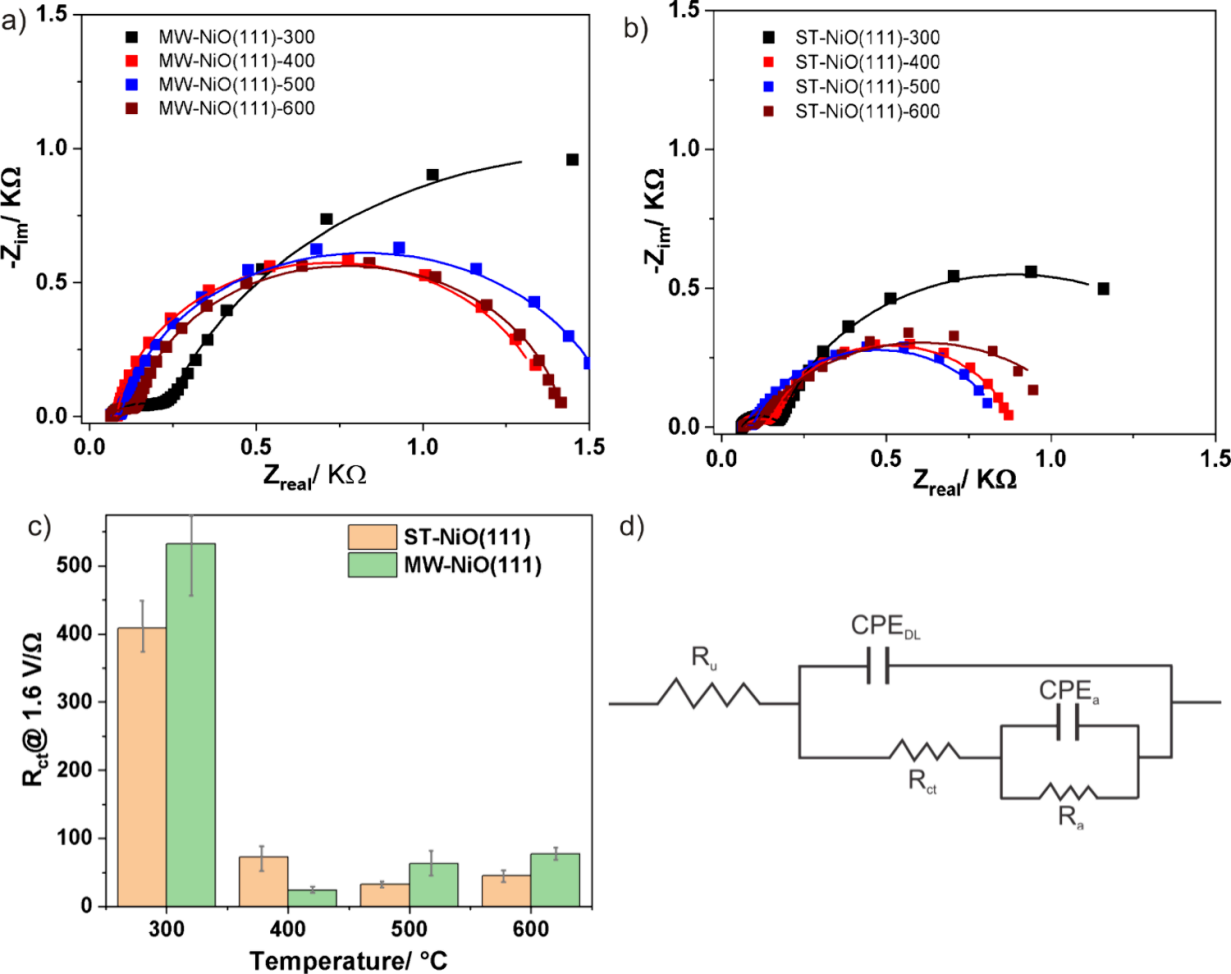
Representative Nyquist plots of NiO(111)-coated GC electrodes in 0.1 MKOH at 1.6 V vs RHE. (a) MW-NiO(111), (b) ST-NiO(111), and (c) *R*_ct_ values extracted from Nyquist plots a and b, and (d) the equivalent circuit model.

Further we conducted EIS analysis at 1.6 V vs RHE, a potential at which the OER reaction begins to occur on most of the tested electrodes (Figure 7a and b). The data were fitted using a circuit model proposed by Watzele et al. (Figure 7d).^63^ The model consists of the uncompensated resistance (*R*_u_), the double-layer capacitance (CPE_cdl_), the charge-transfer resistance (*R*_ct_) and capacitance (CPE_ads_), and the resistance (*R*_ads_) to describe the reversible adsorption of reactive oxygen species. The results suggest that the most active samples, MW-NiO(111)-400 and ST-NiO(111)-500, have the smallest *R*_ct_ values (Figure 7c), proving that these samples facilitate the interfacial charge transfer for the OER reactions.

To investigate the long-term stability of the catalysts, we have performed galvanostatic hold experiments (Figure S18). At a geometric current density of 10 mA cm^–2^, the catalyst layer is detached from the GC electrode after 15 min, and a significant potential jump is observed (not shown). Considering the small catalyst loading (115 μg cm^–2^), such high current density may lead to the formation of high amounts of O_2_ bubbles inside the catalyst layer, resulting in catalyst layer fouling and high resistance.^65^ At a reduced current density of 1 mA cm^–2^, the potentials of MW-NiO(111)-500 and ST-NiO(111)-500 changed by 85 and 70 mV after 5.5 and 9 h, respectively. The ST samples show relatively higher stability under the test conditions. However, the results suggest that the catalyst ink formulation and/or loading need to be optimized for long-term stability at higher current densities.

## 4 Conclusions

In summary, a systematic study of two preparation methods (MW and ST) capable of producing rock salt structured metal oxides with controlled faceting has been adopted and the electrocatalyic activity of electrodes were compared with a loading of 115 μg/cm^2^. A controlled thermal annealing step converts the hydroxides into rock salt NiO nanosheets with hexagonal pores with predominantly (111) exposed crystal facets. Different thermal treatment processes allow tuning the microtextural properties such as the specific surface area and the hexagonal pore dimension and distribution as well as crystalline structure of the materials. The study shows the two synthesis strategies led to materials with very strong structural and morphological similarities. The influence of the annealing temperature was further investigated in the OER activities of electrodes made from these materials. The most OER-active electrode of an MW sample annealed at 400 °C requires an overpotential of 414 mV at 10 mA/cm^2^ with a Tafel slope of 50.8 mV/dec. On the other hand, the ST sample annealed at 500 °C is the most active sample and needs a 405 mV overpotential to achieve 10 mA/cm^2^ possessing a Tafel slope of 52.3 mV/dec. These samples exhibit higher double-layer capacitance values and lower charge transfer resistance for the OER, both of which are attributed to higher OER electrode activities. For both materials, low temperature annealed samples with higher BET areas show low electrode activities. But as the BET area decreases, the electrode activities increase, suggesting an increase in catalytically relevant active surface area or intrinsic activity of the individual active sites. The BET-corrected current density shows that the MW sample still has the potential of higher intrinsic activity if the temperature further increases above 600 °C but at the expense of the geometric current density. ST samples reach their maximal intrinsic as well as geometric activity at 500 °C, and a further increase in temperature deteriorates the activity. The MW samples display a relatively small nanosheet size and have a high density of pores. Additionally, the holes’ edges and corners could have (001) orientation, which is catalytically more active. Being a fast and synthetically direct method, the MW synthesis route has great potential to prepare other faceted materials or extends to the synthesis of doped NiO (111) nanosheets to achieve lower overpotential.

## Supporting Information Available

XRD patterns of α-Ni(OH)_2_ showing the influence of MW synthesis temperature, BJH pore size distribution curves, Raman spectra of ST and MW samples, HRTEM images of as-prepared ST and MW α-Ni(OH)_2_ nanosheets, STEM images demonstrating the hexagonal hole formation and distribution, AFM images estimating the thickness of NiO(111) nanosheets, XPS survey, C 1s and N 1s spectra together with table of fitting parameters and electrochemical measurements including CVs collected at different potential windows, extracted capacitance values, and galvanostatic stability tests (PDF)
