# Supplementary material for: Influence of Annealing Temperature on the OER Activity of NiO(111) Nanosheets Prepared via Microwave and Solvothermal Synthesis Approaches
Source: ACS Appl Mater Interfaces. 2024 Nov 1;16(45):62142–54. doi: 10.1021/acsami.4c14277 (PMC11565572; doi:10.1021/acsami.4c14277)
Supplement: Supplementary file 1 — am4c14277_si_001.pdf [file am4c14277_si_001.pdf]

Supporting information

Influence of annealing temperature on the OER activity of NiO(111) nanosheets prepared via Microwave and Solvothermal synthesis approaches.

Dereje H. Taffa [a*], Elliot Brim[b], Konstantin K. Rücker[a,c], Darius Hayes[b], Julian Lorenz[c*] , Omeshwari Bisen[d], Marcel Risch[d], Corinna Harms[c], Ryan M. Richards[b,e], Michael Wark[a]

[a]*Institute of Chemistry, Chemical Technology I, Carl von Ossietzky University of Oldenburg, Carl-von-Ossietzky-Str. 9-11, 26129 Oldenburg, Germany.*

[b]*Department of Chemistry, Colorado School of Mines, Illinois St. 1500, Golden, Colorado 80401.*

[c]*Institute of Engineering Thermodynamics, German Aerospace Center (DLR), Carl-von-Ossietzky-Str.15, 26129 Oldenburg, Germany.*

[d]*Nachwuchsgruppe Gestaltung des Sauerstoffentwicklungsmechanismus, Helmholtz-Zentrum Berlin für Materialien und Energie GmbH, Hahn-Meitner-Platz 1, 14109 Berlin, Germany*

[e]*Chemical and Material Sciences Center, National Renewable Energy Laboratory, Golden, Colorado 80401.*

*** Corresponding authors**

Dereje.hailu.taffa@uol.de, Julian.Lorenz@dlr.de

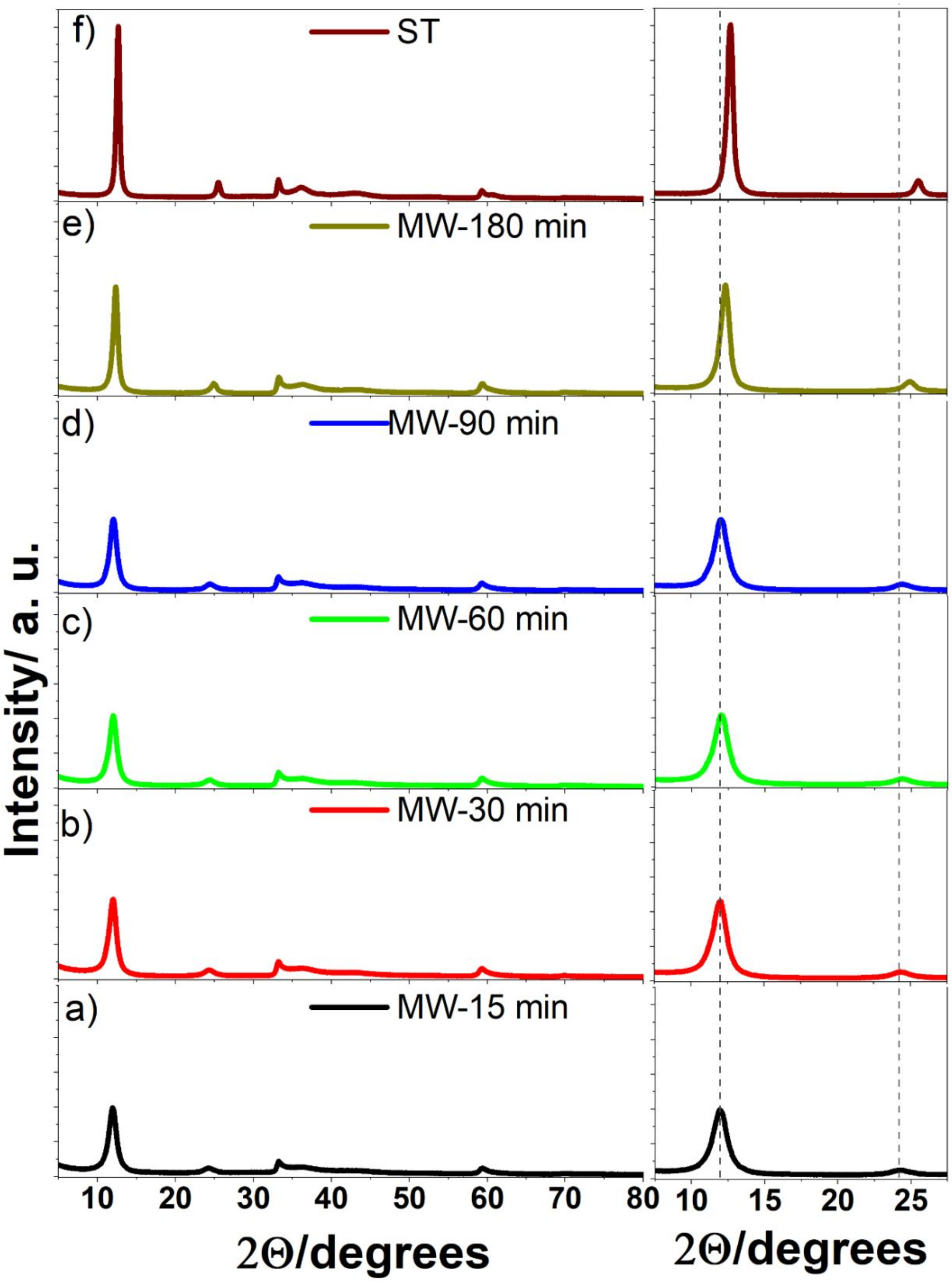

Figure S1: XRD of MW synthesized α-$Ni(OH)_2$ samples (a-e) at 140 ^{o}C showing the effect of synthesis time. f) XRD of ST sample.

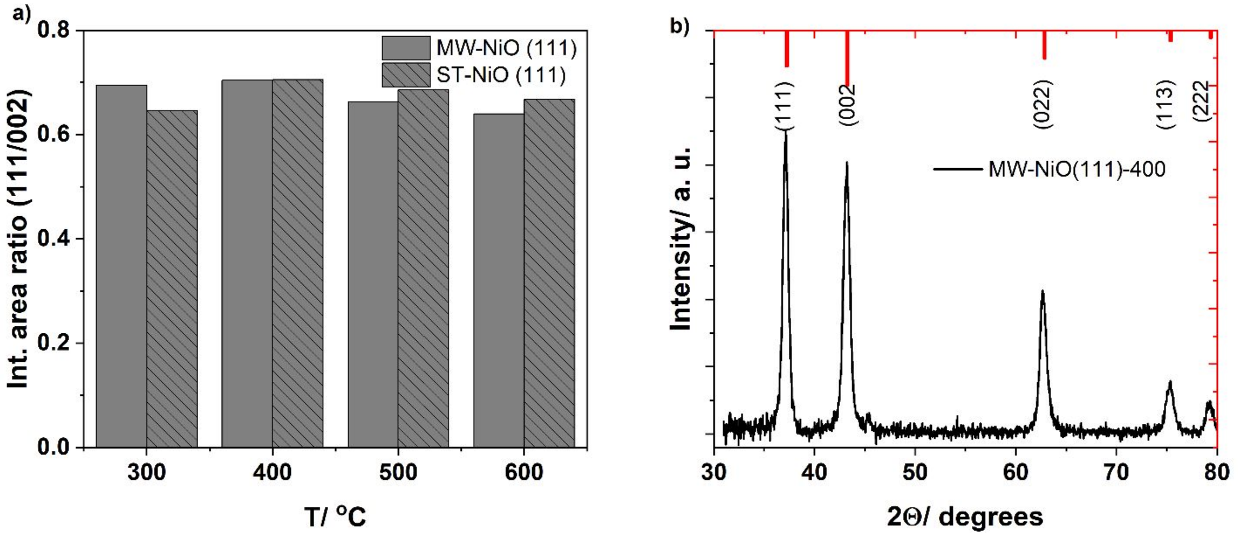

Figure S2: (a) Integral intensity ratios of NiO (111)/NiO (002) as a function of annealing temperature and (b) NiO nanosheets directly grown on a glass substrate with the MW method and annealed at 400 °C with NiO (111) dominant plane.

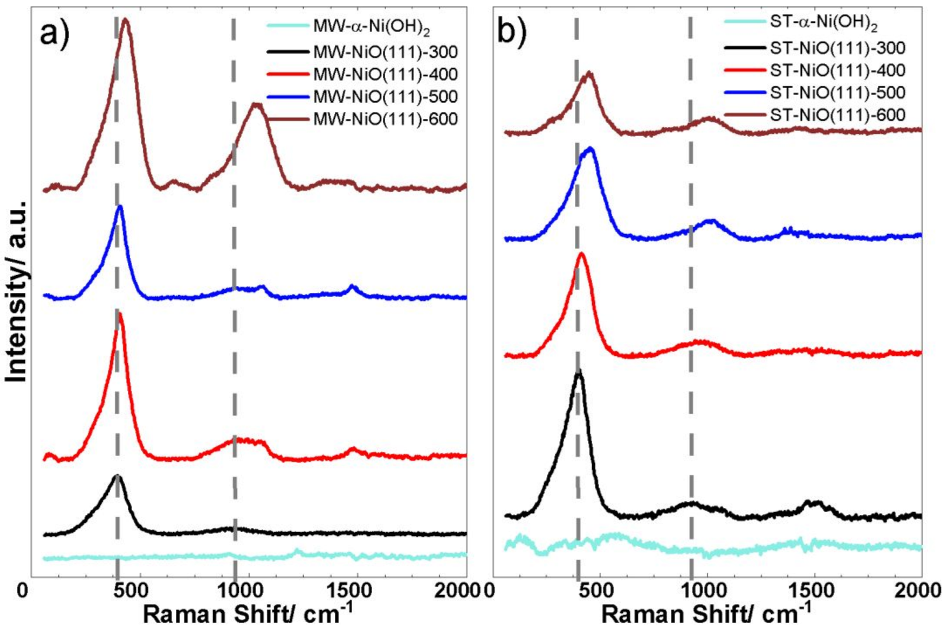

Figure S3: Raman spectra of NiO (111) samples annealed at different temperatures. (a) MW samples and (b) ST samples.

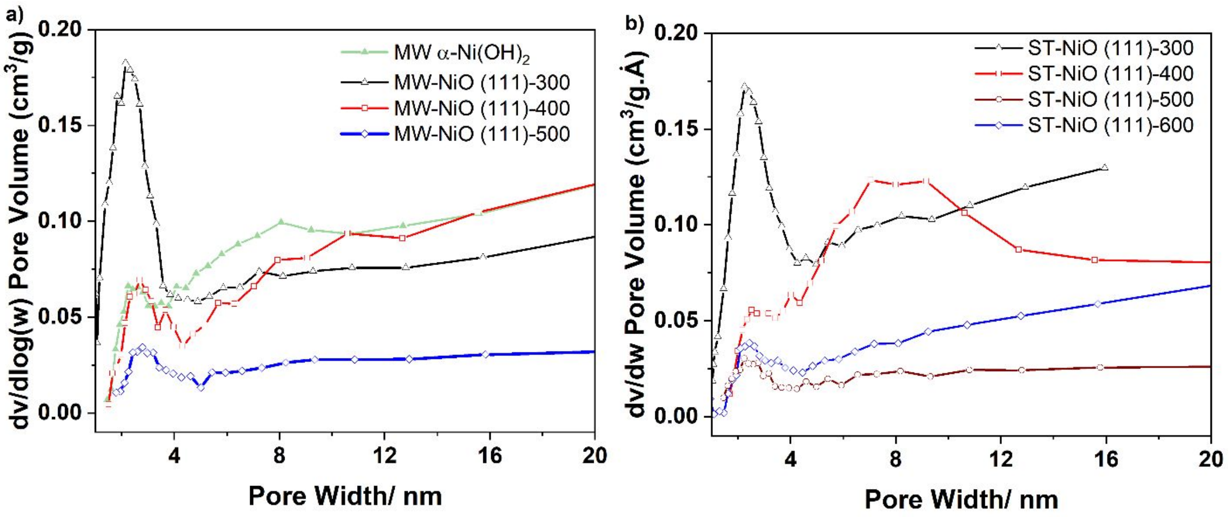

Figure S4: (a) BJH pore size distribution of (a) MW and (b) ST NiO nanosheets as a function of annealing temperature.

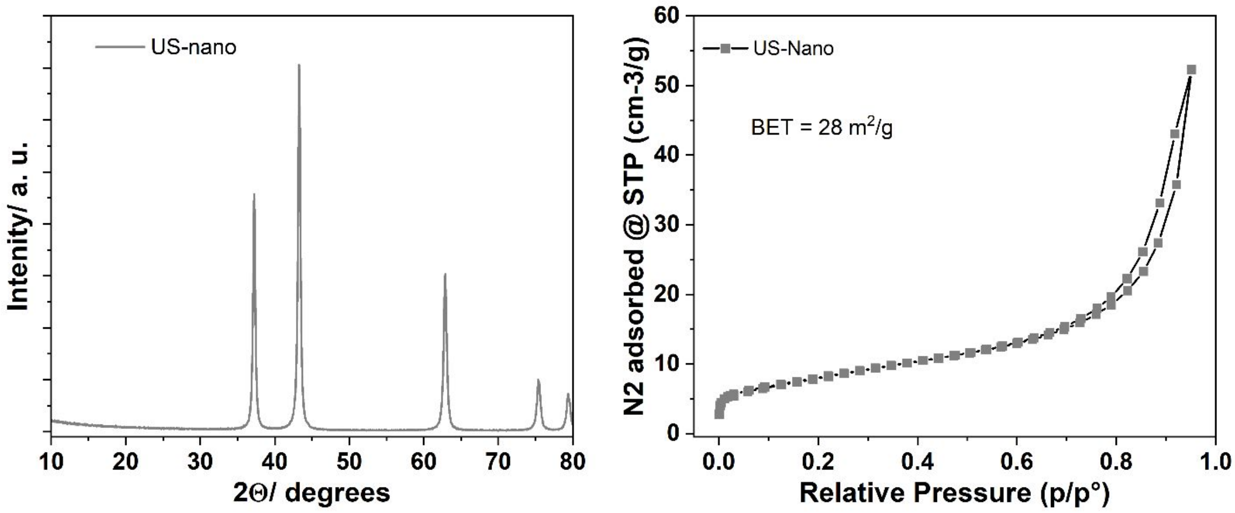

Figure S5: PXRD (left) and N2 gas adsorption (right) measurements for the commercial standard NiO (US- Nano).

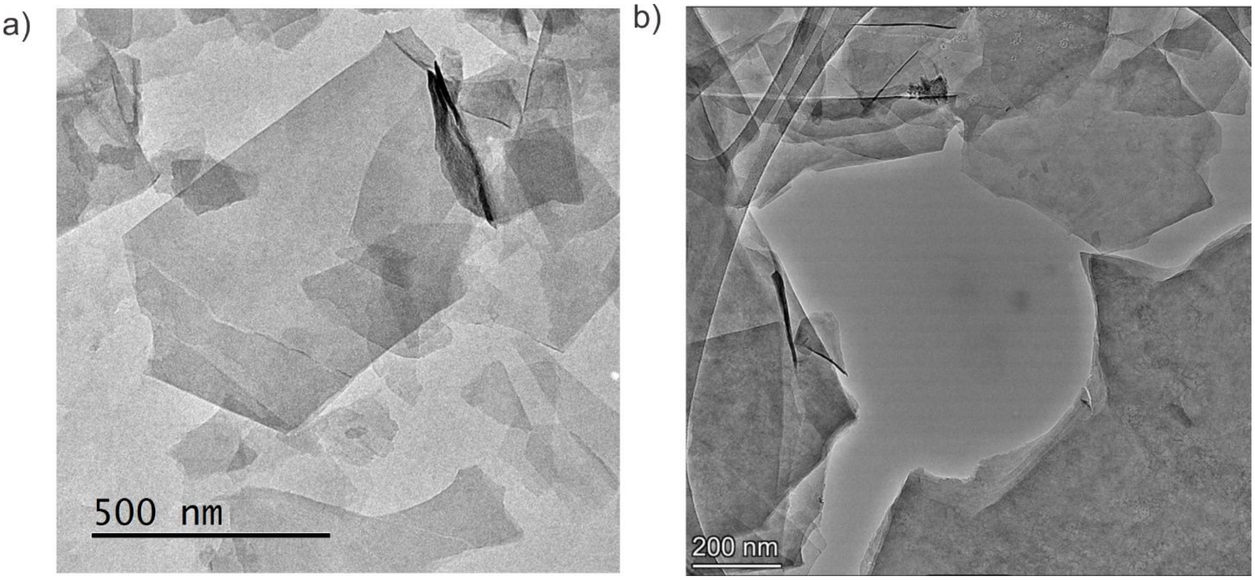

Figure S6: HRTEM of a) MW α-$Ni(OH)_2$ samples synthesized at 140 ^{o}C for 30 min showing micrometer sized nanosheets and b) the ST α-$Ni(OH)_2$ synthesized at 235 ^{o}C for 5hrs.

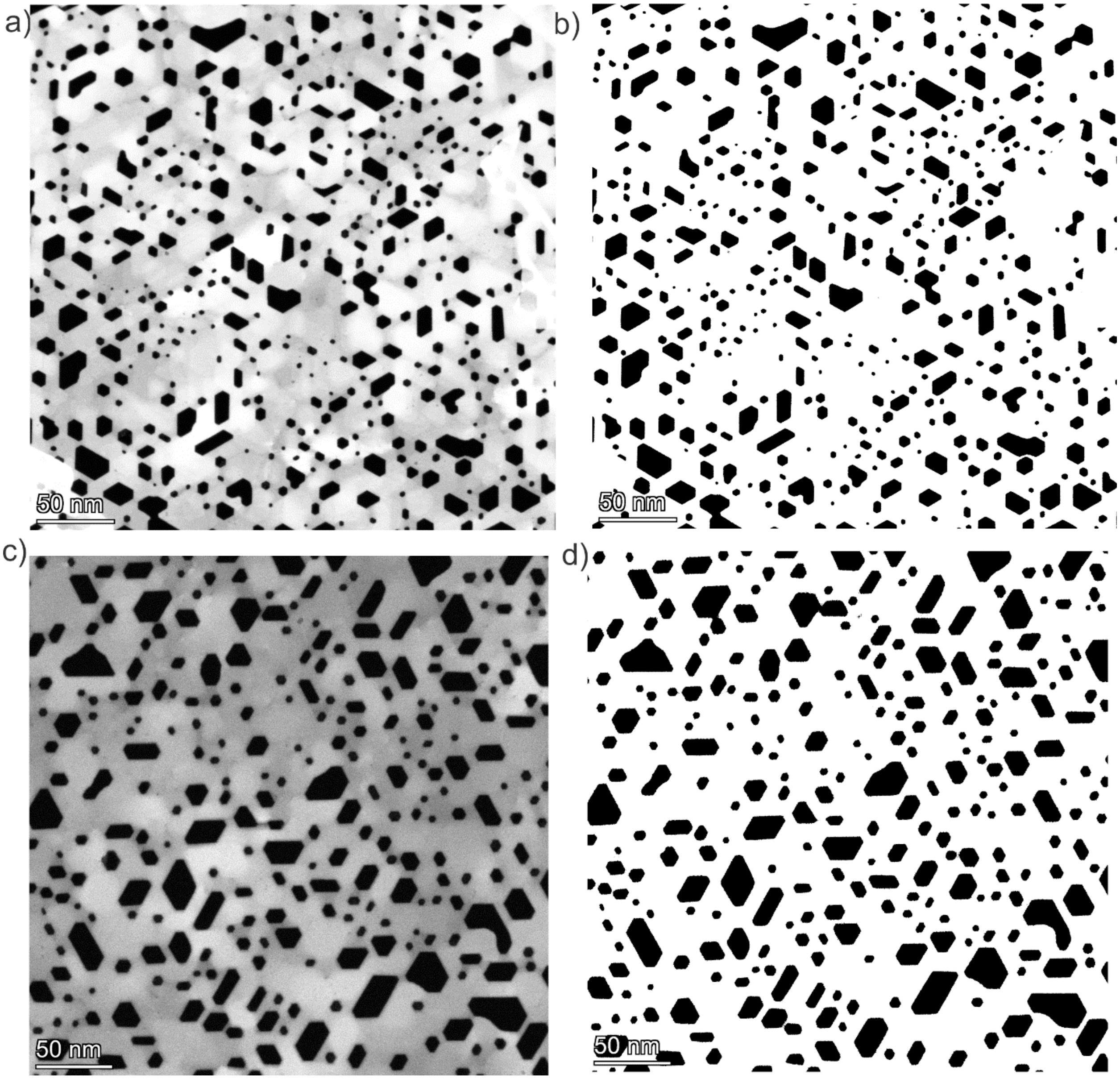

Figure S7: HAADF-STEM images (a) MW-NiO(111)-400 and (c) ST-NiO(111)-400. Image J processed images (b) and (d) showing the size and distribution of the holes.

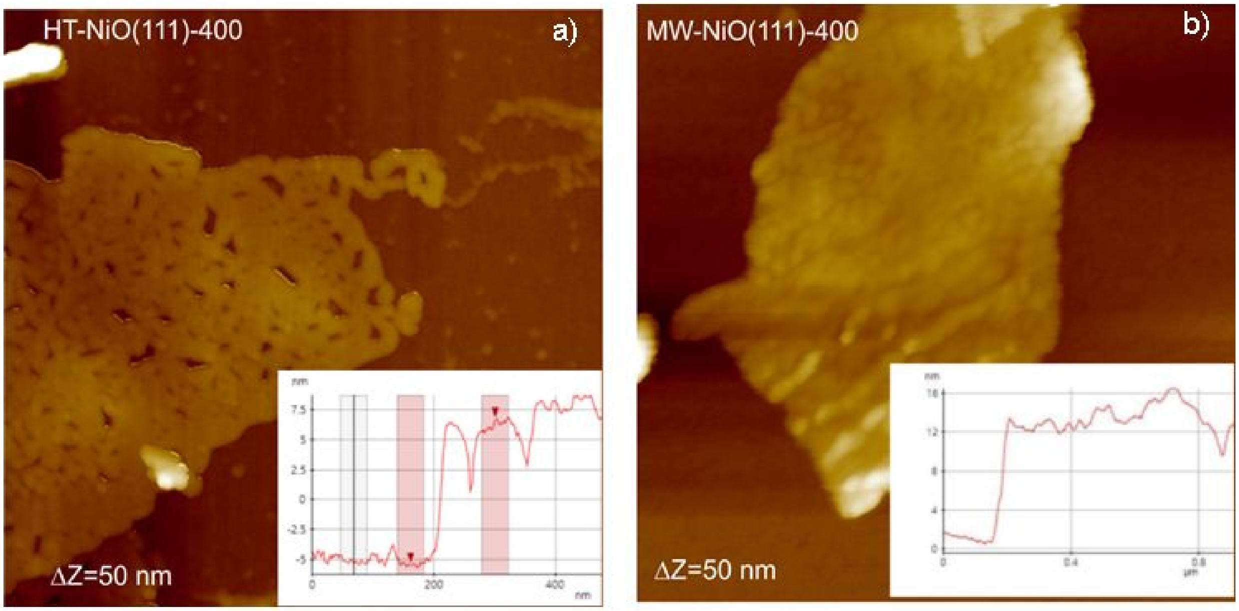

Figure S8: Non-contact mode AFM images of (a) ST sample (1x1 μm^2) and (b) MW sample (1.5x1.5 μm^2) both annealed at 400°C showing the measured thickness.

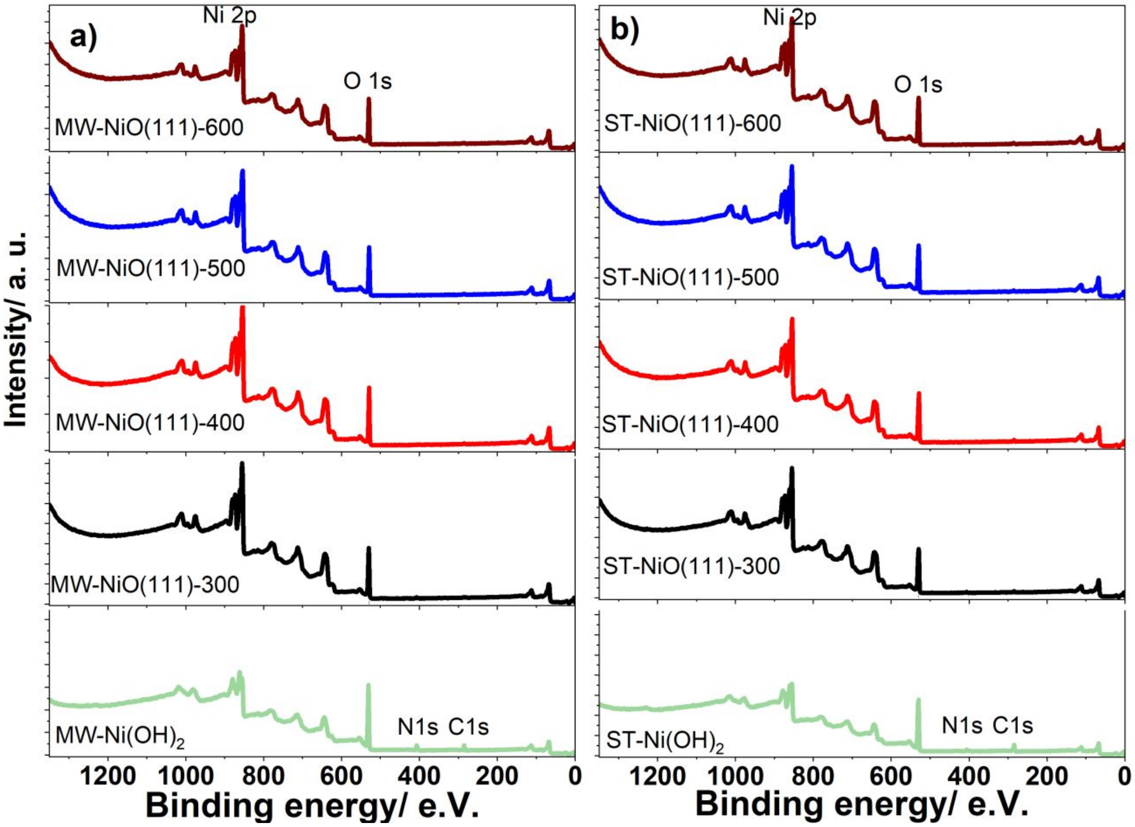

Figure S9: XPS survey spectra of (a) MW samples and (b) ST samples treated at different temperatures showing Ni, O, N and C as the only elements.

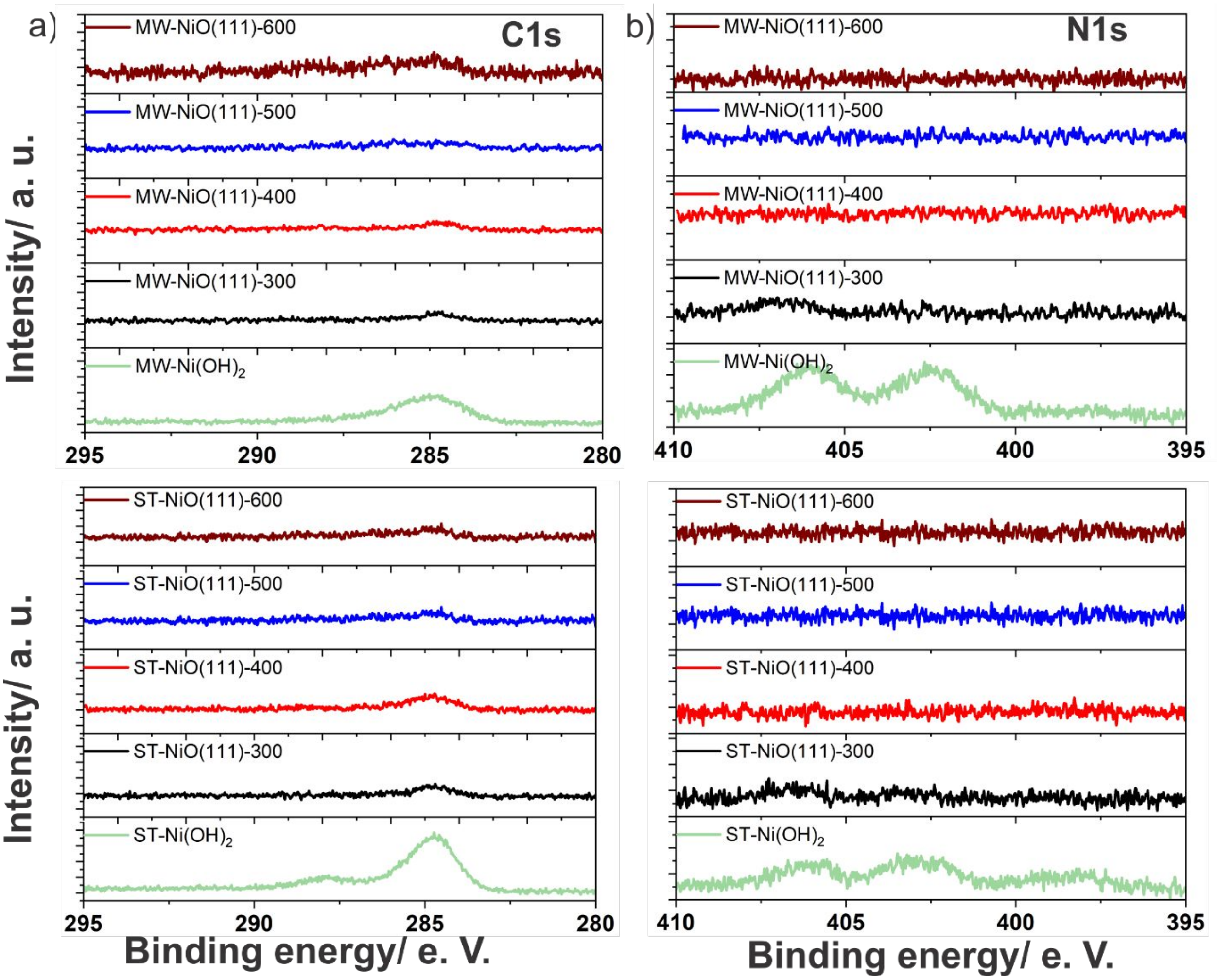

Figure S10: XPS spectra of (a) C1s and (b) N1s for MW and ST samples treated at different temperatures showing relatively high amount of C and N for samples treated at low temperatures.

Table S1: XPS spectra fitting parameters and the Ni:O ratios for MW samples and HT samples treated at different temperatures.

Sample	Assignment	Peaks	Binding energy	FWHM	Ni:O ratio	$O1s_B/O1s_A$
MW-$Ni(OH)_2$	$Ni2p_{3/2}$	A	854.2	2.1		
		B	855.7	2.3		
		C	857.8	2.1		
		S1	860.4	3.0		
		S2	862.9	3.0		
		S3	866.1	2.8		
	O1s	A	529.8	1.6	0.49	0.46
		B	530.9	1.7		
		C	532.2	2.0		
MW-NiO-300	$Ni2p_{3/2}$	A	854.1	1.0		
		B	855.8	3.2		
		S1	861.0	3.4		
		S2	864.0	3.0		
		S3	866.2	3.0		
	O1s	A	529.6	0.9	0.97	0.39
		B	531.44	1.2		
MW-NiO-400	$Ni2p_{3/2}$	A	853.5	1.0		
		B	855.3	3.2		
		S1	860.5	3.5		
		S2	863.5	3.0		
		S3	866.3	2.2		
	O1s	A	529.1	0.9	0.84	0.21
		B	531.0	1.2		
MW-NiO-500	$Ni2p_{3/2}$	A	853.3	1.04		
		B	855.1	1.3		
		S1	859.2	3.35		
		S2	860.8	3.6		
		S3	863.8	3.0		
	O1s	A	528.9		0.82	0.21
		B	530.8			
MW-NiO-600	$Ni2p_{3/2}$	A	853.7	1.1		
		B	855.5	3.2		
		S1	860.7	3.5		
		S2	863.8	2.9		
		S3	865.8			
	O1s	A	529.2		0.85	0.25
		B	531.0			

Sample	Assignment	Peaks	Binding energy	FWHM	Ni:O ratio	$O1s_B/O1s_A$

ST-Ni(OH)$_2$	Ni2p$_{3/2}$	A	854.2	1.94		
		B	855.9	2.3		
		C	858.4	2.4		
		S1	860.4	3.0		
		S2	863.1	3.0		
		S3	865.7	2.78		
	O1s	A	530.1	1.52	0.45	0.28
		B	531.2	1.53		
		C	532.3	1.95		
ST-NiO-300	Ni2p$_{3/2}$	A	853.9	1.0		
		B	855.6	3.2		
		S1	860.8	3.4		
		S2	863.6	3.0		
		S3	866.5	2.5		
	O1s	A	529.5	0.9	0.95	0.38
		B	531.3	1.2		
ST-NiO-400	Ni2p$_{3/2}$	A	853.9	1.0		
		B	855.6	3.2		
		S1	860.8	3.5		
		S2	863.6	3.0		
		S3	866.6	2.4		
	O1s	A	529.3	1.1	0.81	0.25
		B	531.1	1.2		
ST-NiO-500	Ni2p$_{3/2}$	A	853.4	1.04		
		B	855.1	1.3		
		S1	859.5	3.2		
		S2	861.4	3.0		
		S3	864.7	3.0		
	O1s	A	528.9	1.2	0.79	0.21
		B	530.7	1.3		
ST-NiO-600	Ni2p$_{3/2}$	A	853.8	1.1		
		B	855.5	3.4		
		S1	860.7	3.5		
		S2	863.1	3.0		
		S3	865.9	3.0		
	O1s	A	529.3	1.2	0.81	0.25
		B	531.0	1.6		

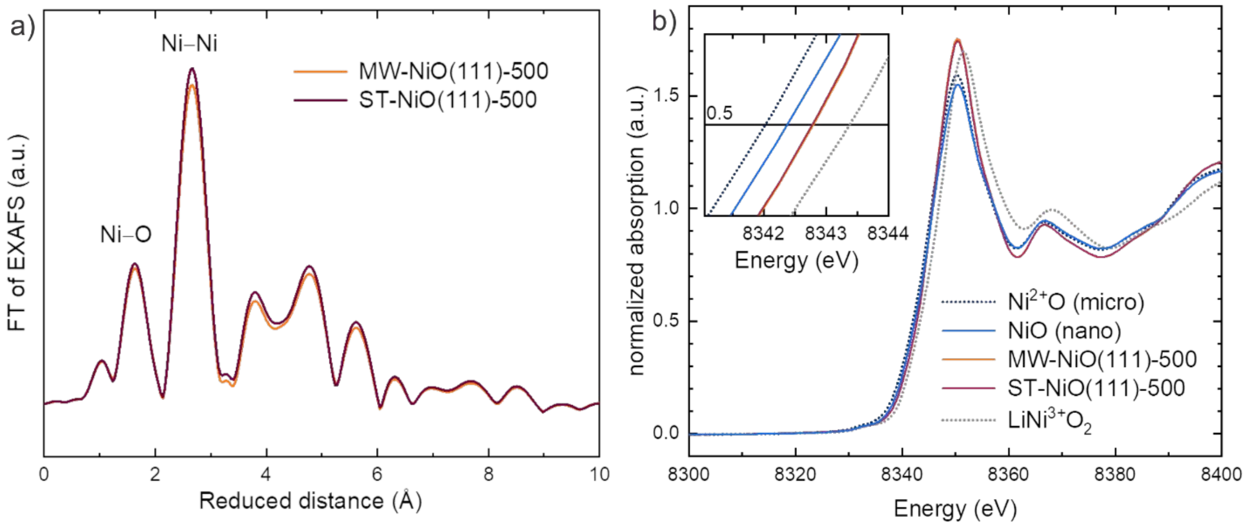

Figure S11: XAS measurements: a) the Fourier transform (FT) of the EXAFS of MW-NiO(111)-500 and ST-NiO(111)-500 and (b) XANES spectra at Ni-K edge showing the edge energies.

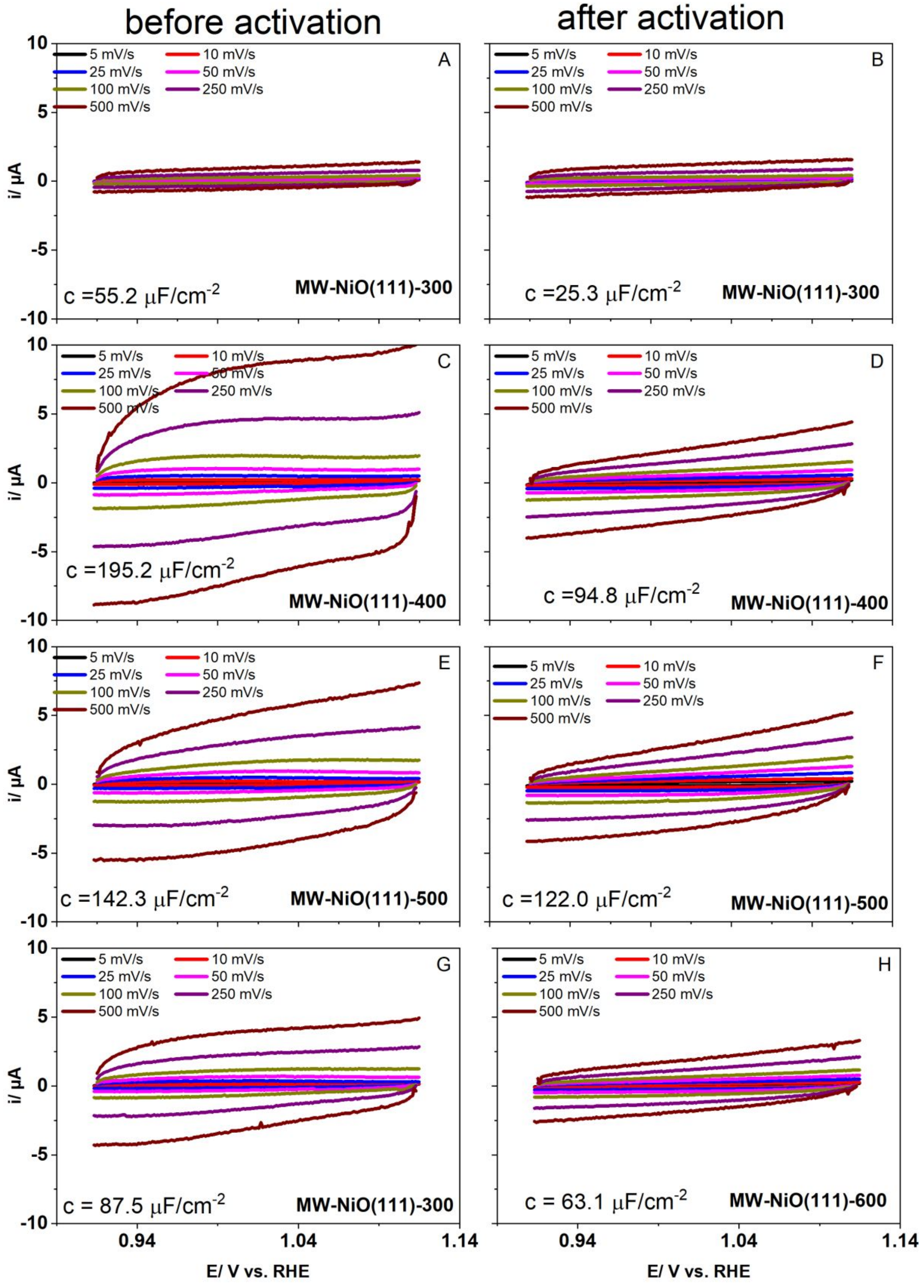

Figure S12: Double layer capacitive curves for the MW-NiO(111) samples before (A, C, E, G) and after activation (B,D, F, H).

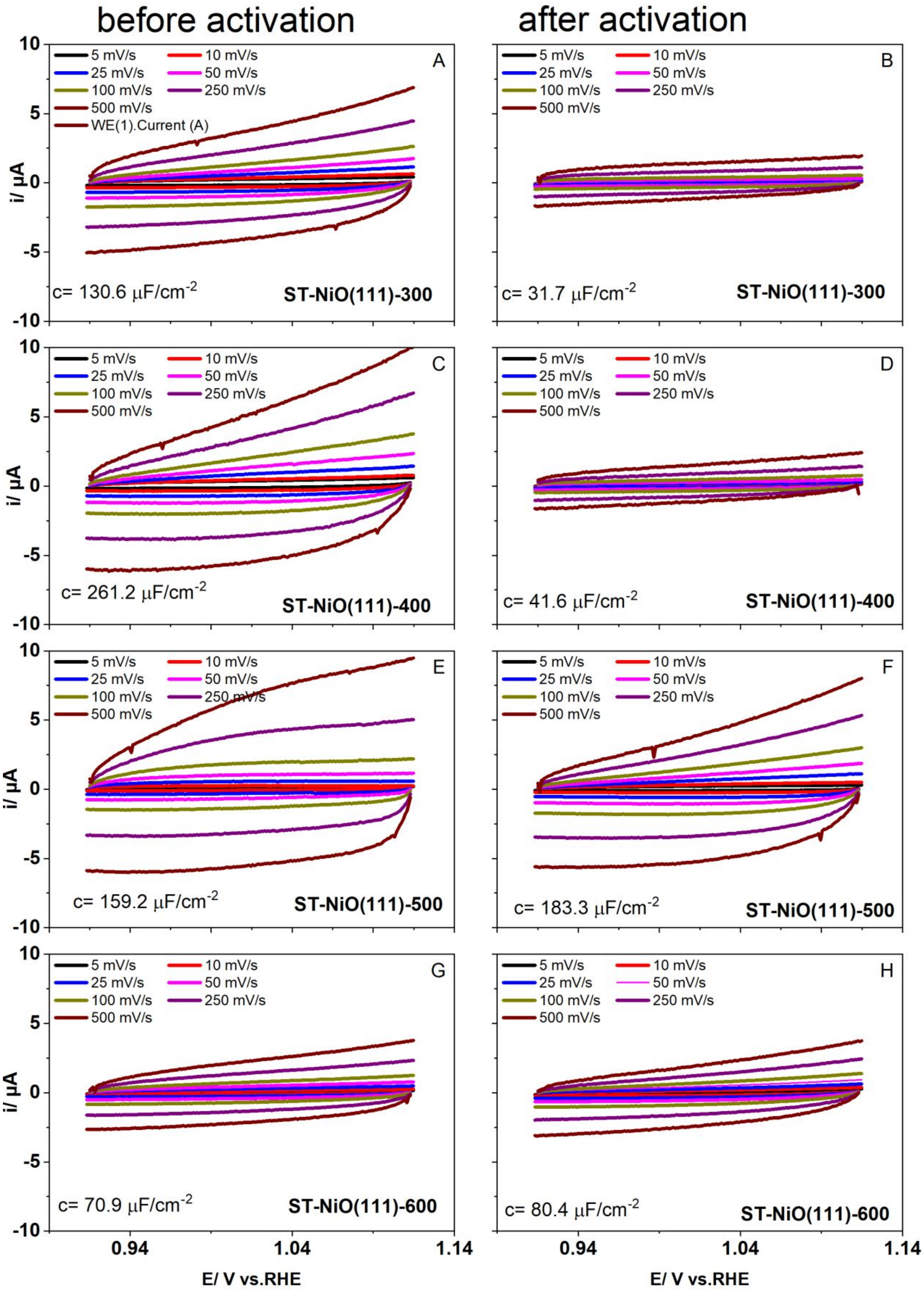

Figure S13: Double layer capacitive curves for the ST-NiO(111) samples before(A, C, E, G) and after activation (B,D, F, H).

.

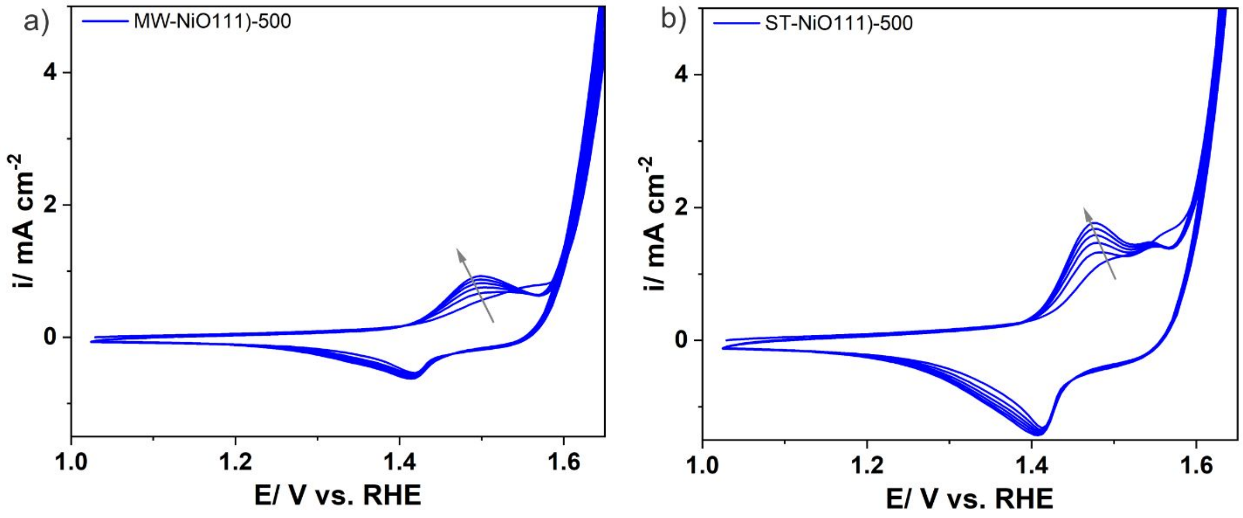

Figure S14: Electrochemical activation step of the NiO (111) nanosheets in 0.1 M M KOH. a) CV measurement of MW-NiO (111) nanosheets and b) ST-NiO (111) nanosheets showing every 10th scan, the scan rate is 100 mV/s. The 1st scan of $Ni^{2+/3+}$ redox transition peak shifted positively compared to the subsequent scans.

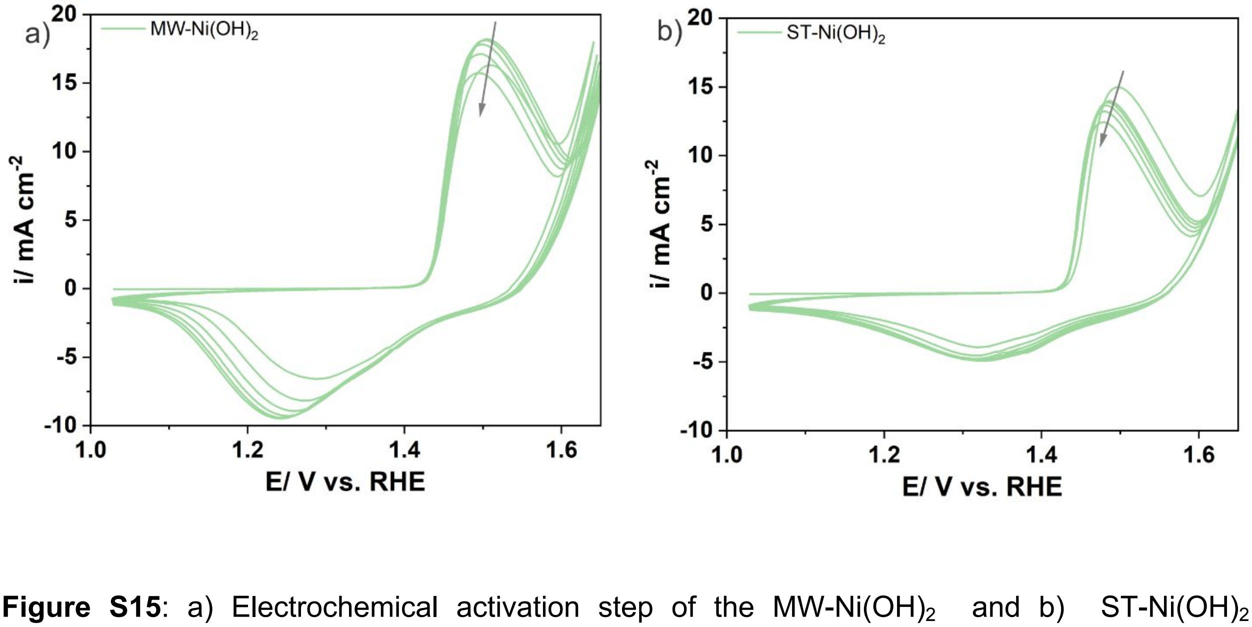

Figure S15: a) Electrochemical activation step of the MW-$Ni(OH)_2$ and b) ST-$Ni(OH)_2$ nanosheets in 0.1 M M KOH. CV measurement showing every 10th scan, the scan rate is 100

mV/s. The 1st scan of $Ni^{2+/3+}$ redox transition peak shifted positively compared to the subsequent scans.

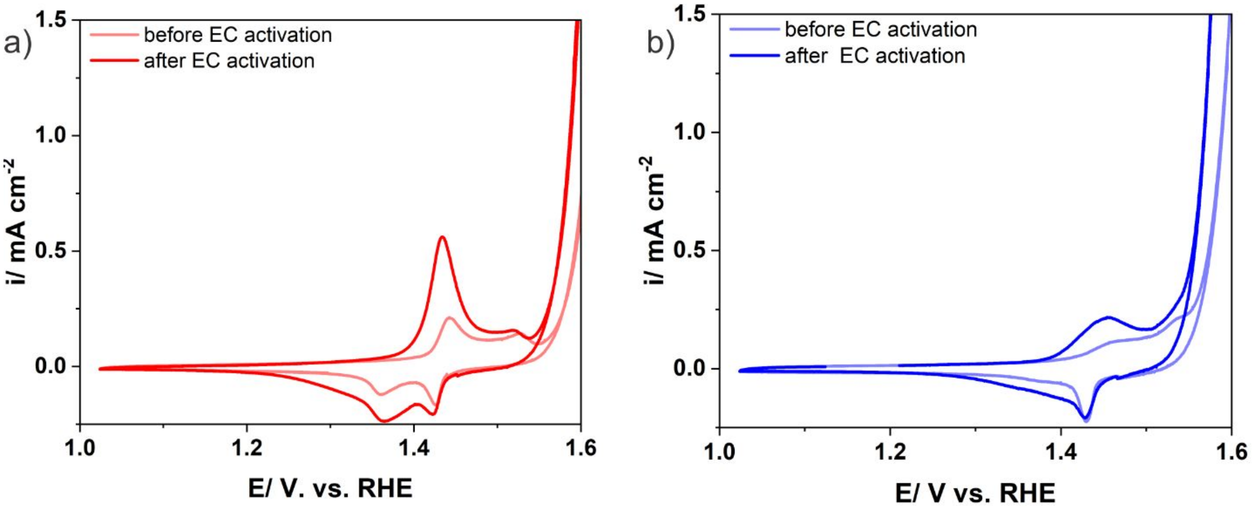

Figure S16: CV showing the activity difference before and after the EC activation step (a) MW-NiO (111) nanosheets annealed at 400 °C and (b) ST-NiO (111) nanosheets annealed at 500 °C. (in 0.1 M M KOH and the CV scan rate of 10 mV/s).

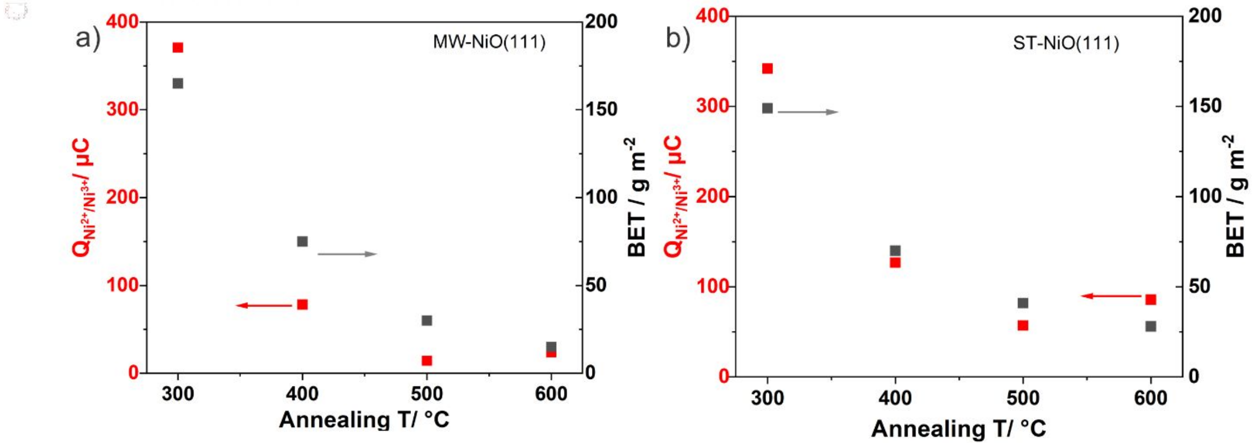

Figure S17: The integral anodic charge under the Ni^{2+}/Ni^{3+} redox peak and the BET area as a function of annealing temperature a) MW-NiO(111) and b) ST-NiO(111).

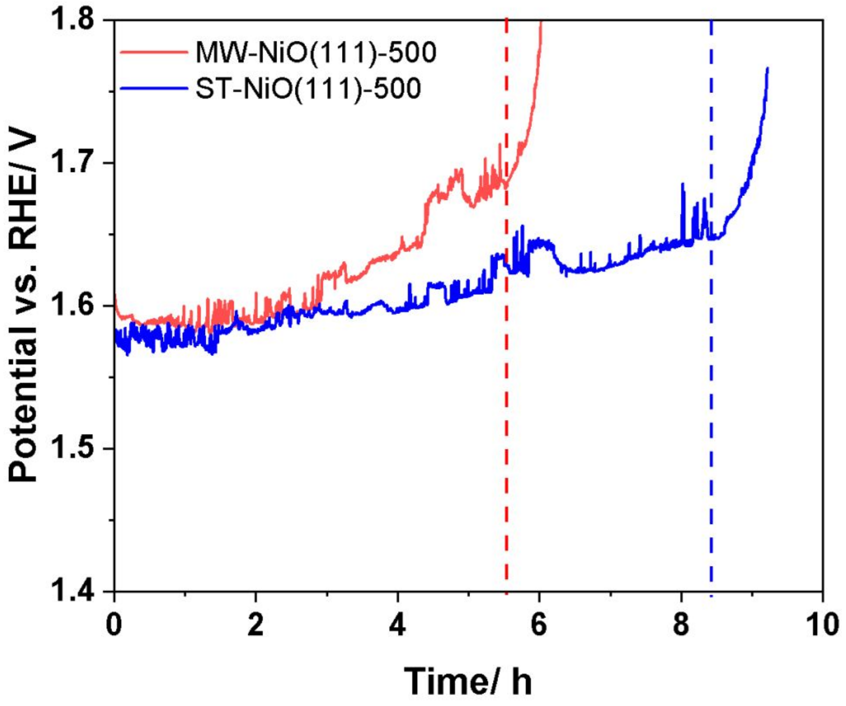

Figure S18: Galvanostatic hold experiments at 1 mA cm^{-2} current density. The broken lines mark start of the catalyst layer detachment.
